# Extensive and diverse lanthanide-dependent metabolism in the ocean

**DOI:** 10.1093/ismejo/wraf057

**Published:** 2025-04-23

**Authors:** Marcos Y Voutsinos, Jillian F Banfield, Harry-Luke O McClelland

**Affiliations:** School of Geography, Earth and Atmospheric Sciences, The University of Melbourne, Melbourne, Victoria, 3053, Australia; Department of Microbiology, Biomedicine Discovery Institute, Monash University, Melbourne, Victoria, 3168, Australia; School of Geography, Earth and Atmospheric Sciences, The University of Melbourne, Melbourne, Victoria, 3053, Australia; Department of Microbiology, Biomedicine Discovery Institute, Monash University, Melbourne, Victoria, 3168, Australia; Innovative Genomics Institute, University of California, Berkeley, CA, 94720, United States; School of Geography, Earth and Atmospheric Sciences, The University of Melbourne, Melbourne, Victoria, 3053, Australia; Department of Structural and Molecular Biology, Darwin Building, University College London, London, WC1E 6BT, United Kingdom

**Keywords:** lanthanides, rare earth elements, dehydrogenases, methylotrophy, methanotrophy, lanthanophores, PQQ

## Abstract

To date, the only known Lanthanide (Ln)-dependent enzymes are pyrroloquinoline quinone-dependent alcohol dehydrogenases. When compared to their Ca dependent counterparts, there is an emerging picture that Ln-dependent versions of these enzymes are generally more efficient, are preferentially upregulated in the presence of Ln when there is functional redundancy, and may even be evolutionarily older. Ln-utilising microbes have furthermore evolved diverse means of solubilizing and acquiring Ln, enabling them to utilize Ln even at trace concentrations. The ocean is the largest dissolved organic carbon pool on Earth, yet the diversity and prevalence of Ln-dependent carbon metabolisms in the ocean is unknown. Here we show that Ln-utilising methanol-, ethanol- and putative sorbose- and glucose-dehydrogenase genes are ubiquitous in the ocean and are highly transcribed, despite extremely low concentrations of Ln in seawater. These enzymes occur in the genomes of 20% of marine microbes, with several individual organisms hosting dozens of unique Ln-utilising enzymes. We found that active microbial methanol oxidation in the ocean is almost entirely Ln-dependent. The widespread biological utility of Ln may help to explain the nutrient-like vertical concentration profiles of these elements in ocean waters and may exert an influence on rare earth element concentration patterns. Microbial Ln-utilisation is a poorly understood component of marine rare earth element biogeochemistry, with potentially important implications for the carbon cycle. The ocean microbiome will be a rich resource for future research into biologically inspired solutions to lanthanide extraction and purification.

## Introduction

The lanthanide series of chemical elements (Lns), which constitute the majority of the rare earth elements (REEs), were long thought to be entirely biologically inert. This view began to change in 2011 with the first hints of lanthanide use in biology [1, 2]. This was soon followed by the discovery of a Ln-dependent methanol dehydrogenase (MDH), employing the redox cofactor pyrroloquinoline quinone (PQQ) [3] and soon after, the discovery of an obligately Ln-utilising bacterium [4]. Several other Ln-dependent dehydrogenases (Ln-DHs) have since been discovered, which have demonstrated activity with methanol [4], ethanol [5], and a range of alcohols and aldehydes [6]. At present, all known Ln-dependent enzymes are PQQ-dependent alcohol dehydrogenases (ADHs). The role of Ln replaces that of calcium, on which all other biochemically characterized enzymes in this subclass depend [7]. Ca-dependent PQQ-dependent quinoproteins have been shown to oxidize a variety of volatile organic substrates and sugars [7–11], which raises the possibility of Ln-dependent versions of these enzymes that would expand the role of Ln in carbon metabolism. In this study, we focus on the ocean, which is the largest dissolved organic carbon (DOC) reservoir on Earth [12], where Ln-dependent metabolism could play a biogeochemically important role.

By far the most intensely studied Ln-dependent enzymes are the MDHs. The Ln-dependent MDHs are divided into five families (XoxF1–5) [13] with Ln coordination confirmed in XoxF1 [14], XoxF2 [4], XoxF4, and XoxF5 [15]. Most (non-methanotrophic) methylotrophs are facultatively methylotrophic and often prioritize growth on multi-carbon compounds (e.g. [16]). When methanol is the preferred available substrate, the presence of Lns has been shown to repress Ca-dependent MDH (*mxaFI)* expression in favor of *xoxF* in microorganisms capable of both Ca- and Ln-dependent methanol oxidation; a mechanism referred to as the “lanthanide switch” [17]. This preference is also reflected in XoxF’s higher substrate affinity compared to MxaFI [4, 18, 19], and higher growth rates on methanol when Lns are available in the environment [18, 19]. While MxaFI oxidizes methanol to formaldehyde, which is then further oxidized to formate by formaldehyde dehydrogenase, some XoxFs have been shown to oxidize methanol to formate in a single step, bypassing the requirement for intermediate enzymes [4, 20]. Lns therefore appear to offer a significant advantage over Ca as cofactors in PQQ-dependent MDH enzymes.

Besides the MDHs, the only other enzymes that have been biochemically confirmed to use lanthanides are found within the PQQ-alcohol dehydrogenase family PQQ-ADH Type 2b. This includes ExaF, an ethanol dehydrogenase from *Methylobacterium extorquens* AM1 [5], which has secondary activities with formaldehyde, methanol, and acetaldehyde, and PedH, from *Pseudomonas alloputida* KT2440, which has demonstrated activity with a variety of short chain alcohols and aldehydes [6]. A greater diversity of Ln-binding PQQ-ADHs was previously predicted from sequence analyses [13]. Lanthanide binding also occurs in small periplasmic proteins involved in trafficking and sorting lanthanides in the periplasm such as lanmodulin [21], its functionally connected interacting partner, landiscernin [22], identified in *M. extorquens* AM1*,* and lanpepsy identified in *Methylobacillus flagellates* [23].

It is unknown how the apparent advantage conferred by Ln-dependent enzymes relates to their prevalence in nature. Whereas Ln-dependent enzymes have been identified in many natural environments [13, 24–28], Ln’s role as a nutrient has been generally assumed to be biogeochemically unimportant and consequently has been omitted from all REE-partitioning models and carbon cycle models. Yet, there is a growing body of evidence that this may be a significant oversight [29]. In the ocean, Lns have long been known to exhibit “nutrient-like” concentration profiles. These observations have previously been explained via scavenging mechanisms, but could also be explained via biological utilisation at the surface and remineralization at depth [30, 31]. Recent work has also shown Ln depletion in seawater related to methane availability, with enhanced removal of the lightest of the lanthanides, which can only be explained by biological uptake [24, 32–34]. Yet Ln-utilising organisms in the ocean face challenges of metal availability that Ca-utilising organisms do not. Ca concentrations in seawater (~10^−2^ M) [9] are both a billion-fold higher and far less spatially and temporally variable than Ln concentrations (~10^−11^ M) [29]. Consequently, some organisms appear to have evolved mechanisms for environmental Ln acquisition where Ln bioavailability is limited by solubility. These mechanisms are poorly understood but are expected to involve active uptake with TonB-dependent transporters (TBDT), analogous to siderophore-mediated iron chelation and transport. Recently, the first lanthanide-binding metallophore, methylolanthanin, was identified in *M. extorquens* AM1. The small organic molecule is produced non-ribosomally by a novel biosynthetic gene cluster including a TBDT, which are highly upregulated when grown in a Ln limiting environment [35].

**Figure 1:**
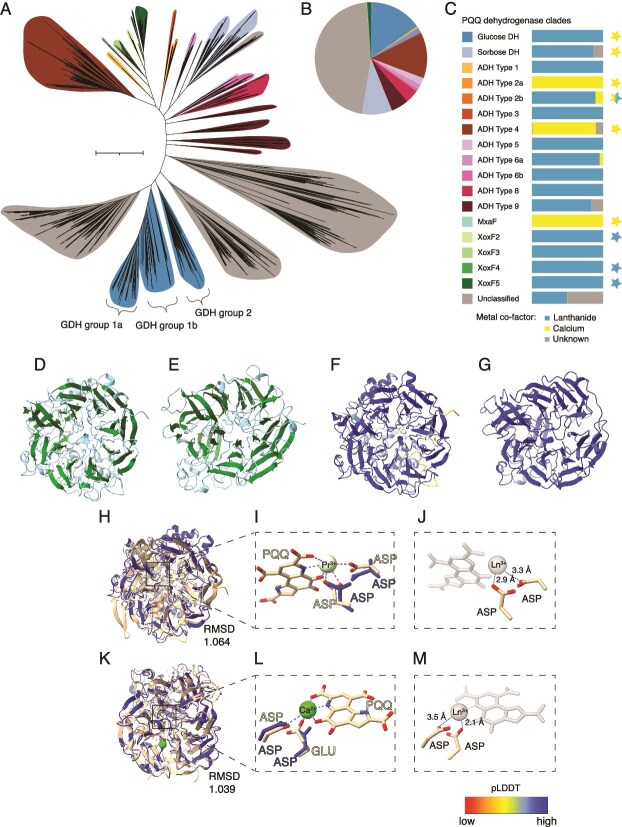
Analysis of 6328 dereplicated PQQ dehydrogenase (PQQ-DH) genes from Tara Ocean metagenomes and metagenome assembled genomes (MAGs). (**A**) Approximately maximum-likelihood tree of PQQ-DH from this study, reference sequences are pruned. For full tree including reference sequences, refer to Fig. S2 and Supplementary Data S1. Scale bar represents 1 substitution per site. (**B**) Pie chart representing the relative abundance of unique PQQ-DHs represented in each clade. (**C**) Legend for A and B. Horizontal bars represent the fraction of sequences in each clade containing lanthanide, calcium or unknown binding domains. PQQ-DH groups with experimentally confirmed representatives are illustrated with a star beside the legend and the fill colour representing their experimentally observed metal dependence: Lanthanide (blue) and/or calcium (yellow). Note, most PQQ-DHs have relatively high levels of substrate promiscuity and overlapping specificities (summarized in Fig. S4). (**D-M**) the two aspartate (ASP) residues required for Ln coordination in PQQ glucose dehydrogenase (GDH) and sorbose dehydrogenase (SDH) are positioned in the active site for Ln interaction. Predicted tertiary structure of (**D**) GDH and (**E**) SDH and their characteristic beta barrel structure (green). Confidence of (**F**) GDH and (**G**) SDH predicted structures as measured by pLDDT. (**H**) GDH aligned with its most similar structure from PDB, PedH, a Ln-dep alcohol dehydrogenase (E-value = 3.35e^−58^, PDB: 6ZCW, in cream). (**I**) Zoomed-in view of the active site showing the ASP residues of GDH and their confidence (blue). The PQQ and residues of 6ZCW are in cream, the green sphere is praseodymium (Pr^3+^), the charges are visualized as negative (red) and positive (blue), and interactions are visualized as purple dashed lines. (**J**) Predicted active site of GDH binding a Ln^3+^ metal cofactor based on its alignment with 6ZCW, black dashed lines represent the interaction distances between ASP residues (cream) and the lanthanide cofactor. The PQQ and metal of 6ZCW are grayed out. (**K**) SDH aligned with its most similar structure from PDB (E-value = 1.74e^−34^, PDB: 4CVB, in cream). (**L**) Zoomed-in view of the active site illustrating the SDH aspartate residues and their confidence (blue), PQQ (cream) and Ca^2+^ (green sphere) of 4CVB. (**M**) Predicted active site of SDH binding a Ln^3+^ metal cofactor based on its alignment with 4CVB, black dotted lines represent the interaction distances between ASP residues of SDH (cream) and Ln^3+^. The PQQ and metal of 4CVB are grayed out.

In this study, using publicly available global metagenomic and metatranscriptomic data, we explore gene distribution, diversity, and levels of transcriptional activity related to Ln-dependent metabolism in the surface ocean. Central to our analysis is a conserved lanthanide binding motif, which was originally proposed based on sequence analysis of crystal structures and homologous sequences identified through genomic and metagenomic data [13, 36], and which we here further support through 3D models of residue geometry and coordination in the active site (Fig. 1I, J, L, M). Our findings reveal that genes encoding Ln-dependent enzymes are ubiquitous and are both more highly transcribed and far more diverse than previously thought, pointing to a central role for Ln in the marine organic carbon cycle. We found, for the first time, that almost all PQQ-glucose and -sorbose DHs in the ocean contain the lanthanide binding motif, indicating lanthanide utilization. We also identify REE transporters and likely lanthanide-chelating metallophones (lanthanophores) in a handful of organisms which will be important biotechnology targets for lanthanide biomining and purification. In addition, Ln-dependent metabolism is strongly related to phosphate concentrations and pH in the modern surface ocean, which suggests a mechanistic coupling between the cycling of lanthanides in the ocean and the marine carbon cycle. The consequences for widespread biological Ln utilisation in the ocean may include interpretations of REE concentration patterns, and a Ln-dependent organic carbon remineralization flux that leaks CO_2_ from the oceanic DOC pool to the atmosphere.

## Materials and methods

### PQQ dehydrogenase identification and classification

We investigated the occurrence of pyrroloquinoline-quinone dehydrogenase (PQQ-DH) enzymes in the ocean by analysing several global datasets: The Tara Oceans metagenomic and metatranscriptomic Ocean Microbial Reference Gene Catalog v2.0 (OM-RGCv2), which includes 83 surface ocean (SO), 53 deep chlorophyll maximum (DCM), and 38 meso-pelagic zone (MES) metagenomic samples, and 103 SO, 49 DCM, and 26 MES transcriptomic samples from the 0.22 to 3 μm size fraction [37] and the set of 1,888 bacterial and archaeal metagenome assembled genomes (BacArcMAGs), which spans all six size fractions of the original Tara oceans metagenomic database [38].

For PQQ-DH identification and classification of families we constructed a phylogenetic tree to discriminate homologous, but functionally distinct proteins that cannot be identified by hidden Markov model (HMM) search alone (Fig. 1). The PQQ-DH sequences were identified in metagenomes using a custom HMM for PQQ-binding dehydrogenases taken from [27]. Across OM-RGCv2, we identified 11,656 PQQ-DH proteins. Proteins greater than 300 amino acids in length were retained and dereplicated at 95% identity using CD-HIT v4.6 [39] resulting in 6,328 PQQ-DH sequences. These sequences were concatenated with a reference set of 2,424 PQQ-DH sequences [27, 40] and aligned using FAMSA [41]. The gaps were then removed from the alignments using trimAl [42] with the parameter -gt 0.1. A phylogenetic tree was constructed using FastTree [43] and sequences were manually classified based on their clustering with PQQ-DH reference sequences and annotated following the previously proposed classifications from Keltjens et al. All phylogenetic trees were constructed using the interactive tree of life (iTOL) [44].

The catalytic binding sites of lanthanide-dependent PQQ-DH enzymes contain an additional aspartate residue absent from the active site of calcium-dependent homologs [36]. Recent studies resolving the metal bound crystal structure have confirmed that the aspartate residue is essential for lanthanide coordination in the active site of XoxF, XoxF1, XoxF2, XoxF4, XoxF5, and PQQ-ADH Type 2b, and that Ln is essential for these enzymes to function (Table S1). In our study, this sequence motif is assumed to be a direct indicator of lanthanide binding, indicating at least facultative, and often obligate, lanthanide dependence of the enzyme [17, 36] (Fig. S1). The multiple sequence alignment of the 6,328 PQQ-DH sequences from this study and 2,424 PQQ-DH reference sequences were used to classify the metal binding domains by manually identifying the aligned amino acid motifs “D-x-A”, “D-x-T”, or “D-x-S" which represent Ca^2+^ coordination or “D-x-D” which represent lanthanide coordination (Fig. S1) [36]. Pyrroloquinoline quinone protein domains were predicted using hmmsearch (version 3.3) [45] to search against the protein family database (version 36.0) [46]. The above method was repeated for the PQQ-DH sequences derived from the dereplicated BacArcMAGs set resulting in 1,956 PQQ-DH sequences. A percent-identity sequence similarity matrix was generated using the 6,328 TARA PQQ-DH amino acid sequences and confirmed PQQ-DHs with Clustal Omega V.1.2.4 [47] and the “—percent-id” function. See supplementary Data S2 for the matrix.

### AlphaFold2 structural and active site modeling of putative glucose- and sorbose-dehydrogenases

The amino acid sequence of OM-RGC.v2.001628136 is classified as sorbose dehydrogenase and OM-RGC.v2.000762315 is classified as glucose dehydrogenase (Fig. 1) and were modeled using AlphaFold2 via ColabFold [48]. The relaxed rank 1 models were visualized using ChimeraX and validated based on their confidence scores as determined by predicted local distance difference test (pLDDT) with only regions with a confidence score of >85 used for analyses. The 3D models generated by AlphaFold2 were used to query the protein data bank (PDB) via Foldseek [49] for their most similar protein structure. The structures were aligned with the AlphaFold2 model using ChimeraX and their active sites analyzed for potential interactions between active site residues and the active site metal cofactor. The PDB ID of the GDH best hit was 6ZCW and SDH best hit was 4CVB.

### Genome phylogenetic classification

**Figure 2:**
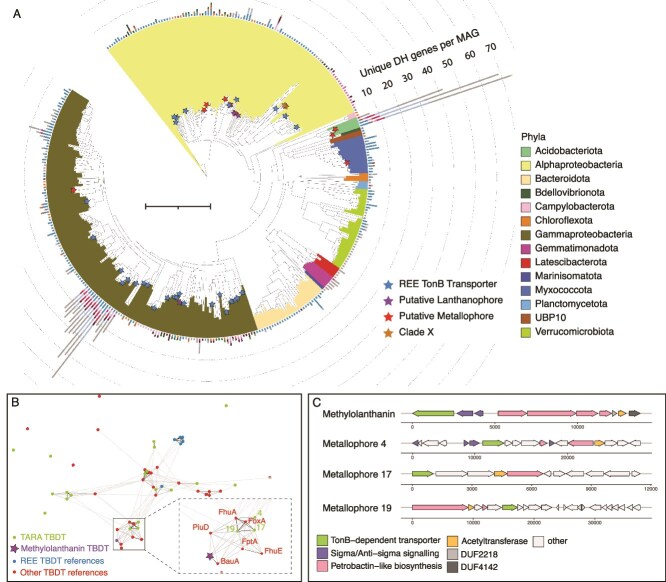
(**A**) Phylogenetic tree of a concatenated alignment of 16 ribosomal proteins from 350 MAGs from SO and DCM, illustrating their number of unique La-PQQ-DH genes, REE outer membrane TonB-dependent transporters (TBDT), putative lanthanophores, and metallophores per genome. MAGs without Ln-binding PQQ-DHs are omitted. An orange star is placed beside Clade X, which contains all three genomes that harbor the most highly expressed Ln-binding PQQ-DH genes (see also Fig. 4B). Note: Colors used in the MAG tree correspond to taxonomic classification and bars around the edge correspond to PQQ-DH clades – Legend in Fig. 1C. Scale bar represents 1 substitution per site. (**B**) Sequence based clustering analysis of the TBDT from 19 metallophores (green dots) detected in TARA genomes with TBDTs of known function and/or structure. PiuD, Fhua, FoxA, FptA, BauA, FhuE, are siderophore transporters. (**C**) Comparison of the biosynthetic gene clusters containing TBDTs that cluster with TBDT from methylolanthanin and putative lanthanophores BGCs from *Thalassospira* [4], *Salinisphaera sp002433465.1* [17], and *Tistrella mobilis* [19] from the Alphaproteobacteria.

To taxonomically classify the microorganisms represented by the BacArcMAGs we used the combination of a concatenated ribosomal protein tree and an rpS3 protein tree (Fig. 2). For the ribosomal protein tree, we searched each genome for 16 ribosomal proteins (RP16) using GOOSOS.py (https://github.com/jwestrob/GOOSOS). The following HMMs were used: Ribosomal_L2 (K02886), Ribosomal_L3 (K02906), Ribosomal_L4 (K02926), Ribosomal_L5 (K02931), Ribosomal_L6 (K02933), Ribosomal_L14 (K02874), Ribosomal_L15 (K02876), Ribosomal_L16 (K02878), Ribosomal_L18 (K02881), Ribosomal_L22 (K02890), Ribosomal_L24 (K02895), Ribosomal_S3 (K02982), Ribosomal_S8 (K02994), Ribosomal_S10 (K02946), Ribosomal_S17 (K02961), and Ribosomal_S19 (K02965). Ribosomal S10 model PF00338 was also used for the identification of *Chloroflexi*. A total of 1,669 genomes containing at least 8 ribosomal proteins were included. The ribosomal protein sequences were then individually aligned using FAMSA and concatenated using the concatenate_and_align.py script from GOOSOS (github.com/jwestrob/GOOSOS/blob/master/Concatenate_And_Align.py). The resulting alignments were stripped of columns containing 90% gap positions using Trimal with the parameter -gt 0.1. A phylogenetic tree was constructed using IQ-TREE [50] and the following settings: iqtree -bb 1000 -nt AUTO -ntmax 48 -mset LG + FO + R. Genomes were then classified at the phylum level using GTDB-TK [51]. Genomes with a completeness below 70% and above 10% contamination were excluded from the analysis.

### Biosynthetic gene cluster and putative lanthanophore prediction

To identify biosynthetic gene clusters (BGCs), antiSMASH 6.0 [52] was run on the BacArcMAGs set using default parameters. Antismash identifies and annotates secondary/specialized metabolite biosynthesis gene clusters in bacterial genomes. Biosynthetic gene clusters retrieved from the genomes were dereplicated using CD-HIT at 95%. Only BGCs on contigs greater than 10 kb were included in the analysis. The antiSMASH tool only classifies BGCs as siderophores when they contain IucA/IucC genes which are specific for aerobactin and aerobactin-like siderophores. To predict the occurrence of siderophores outside of aerobactin we ran two Pfams on the BGCs pfam_transporter20.hmm and all_sbp.hmm to identify the BGCs that contain the transporters: FecCD, Peripla_BP_2, and TonB_dep_Rec. Previous work has shown these transporters are predictive of siderophore activity [53]. Putative lanthanophores were cautiously classified as such when biosynthetic gene clusters with predicted siderophore activity were detected in genomes containing lanthanide dependent enzymes. Geneious was used to visualize the biosynthetic gene clusters [54]. TBDTs were extracted from metallophore BGCs and clustered with a reference sequence set containing TBDTs experimentally confirmed to transport REEs where TBDT mutants prevented REE uptake, (NCBI IDs: WP_060849375.1, WP_235726409.1, WP_244426899.1, WP_245268181.1, WP_003609830.1, UNIPROT ID: A0A4P9UKZ7), biochemically characterized TBDTs (taken from TCDB database), and *M. extorquens* AM1 methylolanthanin TBDT (*mluA,* META1p4129). Sequences were clustered based on BLASTp all-against-all searches using CLANS [55] with an E-value cutoff of 1x10^−10^ until equilibrium. The BGC genes were annotated against CDD V3.19 [56], the Pfam protein family database v34.0 [46] and the NCBI RefSeq protein database release 202 [57].

### Biogeography of PQQ-DH genes and transcripts in the global ocean

**Figure 3:**
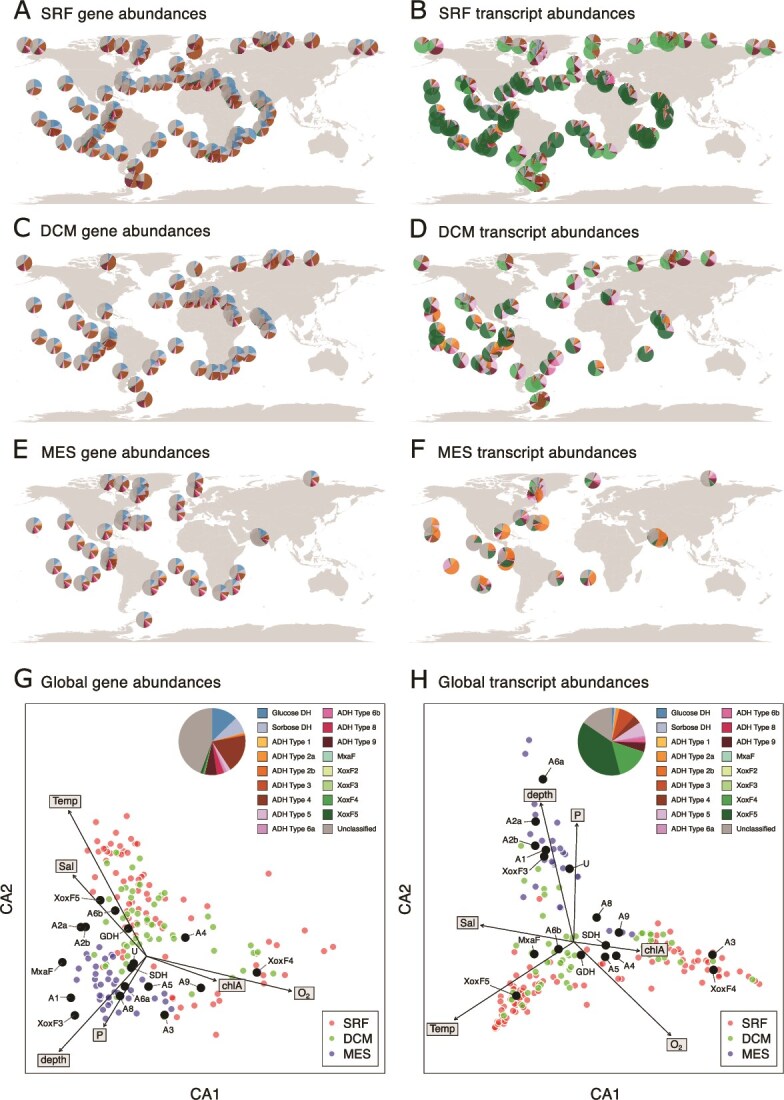
(**A**) Distribution of PQQ DH gene abundances throughout the surface ocean in community metagenomes. (**B**) Distribution of PQQ DH transcript abundances throughout the surface ocean in community metatrascriptomes. (**C**) Distribution of PQQ DH gene abundances throughout the deep chlorophyll maximum in community metagenomes. (**D**) Distribution of PQQ DH transcript abundances throughout the deep chlorophyll maximum in community metatranscriptomes. (**E**) Distribution of PQQ DH gene abundances throughout the mesopelagic zone in community metagenomes. (**F**) Distribution of PQQ DH transcript abundances throughout the mesopelagic zone in community metatrascriptomes. (**G**) Global gene abundances (pie chart, inset), and correspondence analysis showing how relative abundances of genes (black dots) vary with environmental variables (arrows). In the point labels, “ADH type” is abbreviated to “A” for clarity. (**H**) As G but transcript rather than gene abundances.

The abundance of PQQ-DH genes from the global ocean were estimated by analysing the OM-RGC-v2 [37] and BAC_ARC_MAGs dataset [58] (Fig. 3). The analysis was conducted using the Ocean Gene Atlas platform [59] with an expected threshold of 1E^−10^. The abundances were calculated as percent of total coverage using the reads per kilobase per million mapped (RPKM) method. The abundances of PQQ-DH transcripts were also estimated using the above methods. Only sequences with a complete cofactor binding domain were included in this analysis (calcium, lanthanide, or unknown). Sequences with an incomplete cofactor binding domain were excluded.

### Ordinations

The metagenomic and transcriptomic datasets of PQQ-DHs were explored in the context of their chemical metadata (Fig. 3). From these metadata we chose only those variables with high quality and relatively complete datasets. These variables were: temperature, salinity, chlorophyll A concentrations, depth, oxygen concentration, and total phosphate. We excluded nitrate from the analysis owing to its high collinearity with phosphate. Figures 3G and H present the output from an unconstrained correspondence analysis, which was chosen to emphasize relative differences in gene abundance and expression between sites (as opposed to an unscaled analyses which is dominated by the most abundant genes / transcripts) and to avoid issues associated with non-linearity in the relationship with explanatory variables.

### Phylogenetic classification of clade X

**Figure 4:**
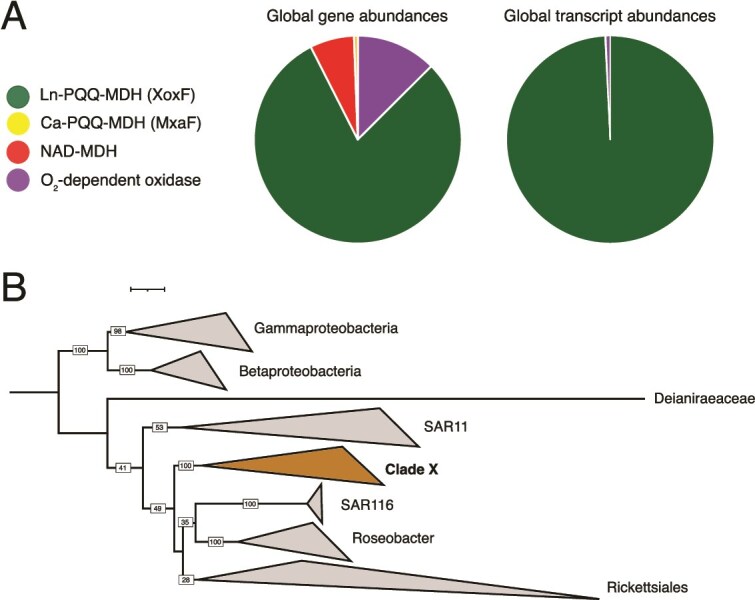
Prevalence of PQQ MxaF, PQQ XoxF, NAD MDH, and O_2_ M-oxidase in the global ocean (**A**) gene abundances and transcript abundances in the combined surface ocean, deep chlorophyll maximum and mesopelagic. PQQ MxaF, PQQ XoxF, and NAD MDH abundances were derived from the 0.22–3 μm size fraction and O_2_ MDH abundances were derived from the 0.8–2000 μm size fractions. (**B**) Phylogenetic tree showing the placement of clade X.

To identify the dominant Ln-utilising organisms a phylogenetic tree containing the most highly expressed *xoxF* genes from the metatranscriptomes with all *xoxF* genes identified in the MAGs was created (Fig. S5). Three genomes from the Alphaproteobacteria (TARA_PSW_86_MAG_00242, TARA_MED_95_MAG_00188, TARA_MED_95_MAG_00017), previously classified as *Rickettsiales* using GTDB-Tk and CheckM [60], contained the most highly expressed *xoxF5* sequences. They did not contain 16S sequences so they were classified based on the phylogenetic placement in a ribosomal protein tree with reference genomes taken from Luo 2015 [61], Delmont et al 2022 [38] and Schön et al 2022 [62] (Fig. 4). Each genome was searched for 16 syntenic ribosomal marker proteins (rp16) using GOOSOS.py (https://github.com/jwestrob/GOOSOS). Genomes that contained at least 8 of 16 rp16 genes were retained and individually aligned using FAMSA and concatenated using the concatenate_and_align.py script from GOOSOS (https://github.com/jwestrob/GOOSOS/blob/master/Concatenate_And_Align.py). The multiple alignment was trimmed for phylogenetically informative regions using BMGE (−m BLOSUM30) [63] and a maximum-likelihood tree was inferred using IQ-TREE [50] and the following parameters: -bb 1000 -st AA -m MFP.

### Methane monooxygenase phylogenetic analysis

The presence of aerobic methanotrophy is typically investigated using the *pmoA* gene, which encodes subunit A of particulate methane monooxygenase (pMMO) and *mmoX*, which encodes subunit A of soluble methane monooxygenase (sMMO). The following HMMs were used to search the metagenomes and MAGs: K10944 (pmoA) and K16157 (mmoX). Two phylogenetic trees were made to confirm the retrieved protein sequences. For each tree the sequences were concatenated with references taken from Singleton et al. [64] and individually aligned using FAMSA [41] with default settings. The gaps were then removed from the alignment using trimAl [42] with the parameter -gt 0.1. An approximately maximum-likelihood tree was constructed using FastTree [43] and the WAG + GAMMA parameters and sequences were manually classified based on their relationship with the MMO reference sequences. Both phylogenetic trees were constructed and annotated using iTOL [44]. See Supplementary Data S3 for the phylogenetic tree.

### NAD MDH identification and classification

We investigated the occurrence of nicotinamide methanol dehydrogenase (NAD MDH) genes in the global ocean by analyzing the metagenomic and metatranscriptomic Ocean Microbial Reference Gene Catalog v2.0 (OM-RGCv2) [37]. For NAD MDH identification we constructed a phylogenetic tree to discriminate homologous, but functionally distinct proteins that cannot be identified by HMM search alone. The NAD MDH sequences were identified using K00093.HMM. Across OM-RGCv2, we identified 9,043 NAD MDH proteins. Protein sequences with lengths between 320 and 420 amino acids were retained and dereplicated at 95% identity using CD-HIT v4.6 [39] resulting in 3,766 NAD MDH sequences. The sequences were concatenated with a set of experimentally confirmed NADH MDH sequences taken from [65] and aligned with FAMSA [41]. The gaps were then removed from the alignments using trimAl [42] with the parameter -gt 0.1. The sequences were manually inspected and those that contained the NAD and metal binding domains were retained, resulting in a set of 2,602 NAD MDH homologs. A phylogenetic tree was constructed using FastTree [43] and sequences were manually classified based on their relationship with the NAD MDH reference sequences. See Supplementary Data S4 for the phylogenetic tree.

### Oxygen dependent methanol dehydrogenase identification and classification

We investigated the occurrence of oxygen dependent methanol dehydrogenase (O_2_ MDH) genes in the global ocean by analyzing the metagenomic and metatranscriptomic Marine Atlas of Tara Oceans Unigenes (MATOU) collected during the Tara Oceans expedition [66]. This dataset was constructed to target the eukaryotic fractions of the metagenomes, including enrichment for eukaryotes and excluding prokaryotes. We present a direct comparison between O_2_ MDH from this dataset and prokaryotic MDHs from the metagenomes / metatranscriptomes which targets free-living microbes (Fig. 4). This comparison therefore excludes potential prokaryotes in size fractions greater than 3 μm, which might occur in association with larger particulates or in the guts of eukaryotes.

For O_2_ MDH identification we constructed a phylogenetic tree to discriminate homologous, but functionally distinct proteins that cannot be identified by only HMM search. The O_2_ MDH sequences were identified using the K17066.HMM profile. We identified 5,491 O_2_ MDH proteins. Protein sequences with lengths between 190 and 700 amino acids were retained and dereplicated at 95% identity using CD-HIT v4.6 [39] resulting in 3,197 O_2_ MDH sequences. The sequences were concatenated with a set of O_2_ MDH sequences taken from [67] and aligned with FAMSA [41]. The gaps were then removed from the alignments using trimAl [42] with the parameter -gt 0.1. A phylogenetic tree was constructed using FastTree [43] and sequences were manually classified based on their relationship with the O_2_ MDH reference sequences. For the phylogenetic tree see Supplementary Data S5.

### Biogeochemistry

**Figure 5:**
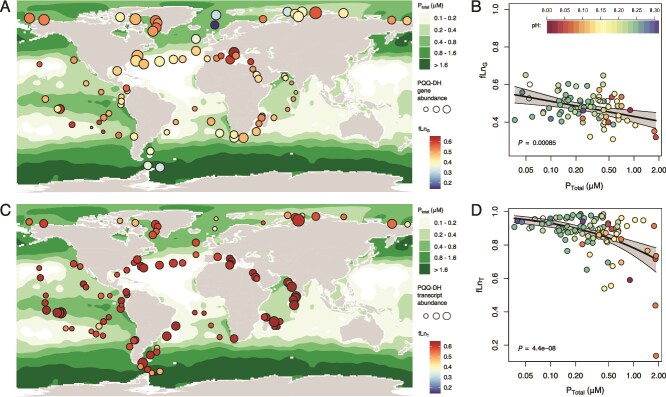
Spatial distribution of Ln-utilising PQQ DHs in the surface ocean. (**A**) Fraction of PQQ DH genes present that contain the Ln-binding motif (fLn_G_), plotted over annual average total phosphate concentrations. (**B**) fLn_G_ plotted against the annual average total phosphate concentration at the location of sampling. (**C**) Fraction of PQQ DH transcripts present that contain the Ln-binding motif (fLn_T_), plotted over annual average total phosphate concentrations. (**D**) fLn_T_ plotted against the annual average total phosphate concentration at the location of sampling. In B and D trendlines are logistic regression models, and p values correspond to the x-axis coefficient.

Phosphate concentrations (Fig. 5) are taken from a well-established interpolated dataset of monthly average phosphate (WOA2018, NOAA), and pH was calculated from temperature, salinity, total alkalinity and DIC taken from the CO2OceanSODA-ETHZ data set [68], for the period Jan 2018 to December 2018, at 1 degree spatial resolution. To compare phosphate concentrations and pH with the metagenomic / metatranscriptomic sequence data we took the coordinates and date of the site of collection, and averaged annual averages in ocean chemistry from grid squares within a 1-degree radius of the collection site. Correlations between the fraction of DH genes that contained the Ln-binding motif (fLnG) and between the fraction of DH transcripts that contained the Ln-binding motif (fLnT) and average annual total phosphate concentrations were quantified using logistic regression analysis.

**Figure 6:**
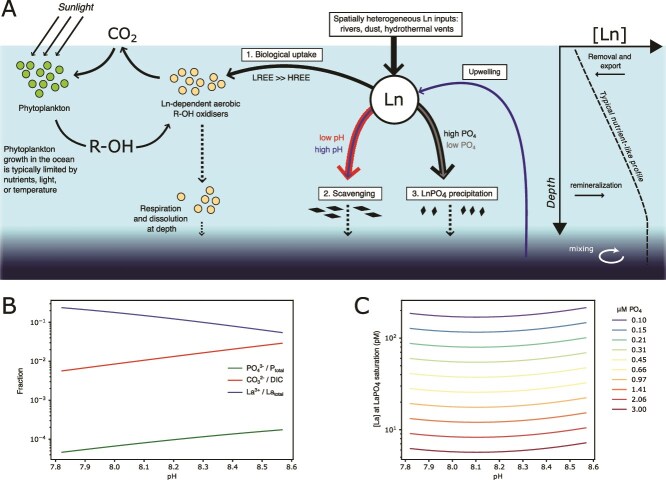
(**A**) Schematic for the proposed coupling between the carbon cycle and lanthanide cycling. (**B**) Representation of phosphate, DIC, and La (as representative of Ln) speciation with pH under typical surface ocean conditions. Note La^3+^ / La_Total_ increases strongly with decreasing pH. (**C**) Total La concentrations at LaPO_4_ saturation. Where La concentrations are higher than this value, the precipitation of LaPO_4_ is thermodynamically favored. The concentration of La at LaPO_4_ saturation is only weakly impacted by pH, but is strongly controlled by total phosphate concentrations. In both plots pH is varied by changing total dissolved inorganic carbon at constant alkalinity.

The aqueous chemical model was developed using the python implementation of Reaktoro (reaktoro.org), using the phreeqc llnl database (thermodynamic data compiled by the Lawrence Livermore National Laboratory), and HFK activity model. A solution consisting of elements H, O, Na, Cl, C, Mg, K, S, Ca, La, and P with typical seawater concentrations of ionic species: 0.468 mol / kg Na^+^, 0.546 mol / kg Cl^−^, 0.0533 mol / kg Mg^2+^, 0.0281 mol / kg SO_4_^2−^, 0.0023 mol / kg HCO_3_^−^, 0.0104 mol / kg Ca^2+^, 0.00997 mol / kg K^+^, 20 x 10^−12^ mol / kg total La, and 10^−6^ mol / kg PO_4_^3−^ was speciated at a temperature of 15°C and 1.0 bar pressure. LaPO_4_ was found to be the only La bearing mineral phase occurring in non-negligible amounts. Simulations involved changing CO_2_ at constant alkalinity. Increases in CO_2_ leads to acidification and carbonation of seawater, both of which impact Ln speciation. As pH of the water decreases, the fraction of P in the form of free PO_4_^3−^ and the fraction of DIC in the form of CO_3_^2−^ decrease. A decrease in PO_4_^3−^ decreases the saturation state of LaPO_4_, however a decrease in CO_3_^2−^ decreases the degree of CO_3_^2−^ complexation of La, and therefore the fraction of La in the form La^3+^ increases (Fig. 6). These effects are counteractive for solubility of LaPO_4_ with a minimum at around pH 8.1, but very little change with changing pH for a realistic range of ocean conditions (Fig. 6). We conclude that the effect of pH change at constant alkalinity will likely have a significant effect on adsorptive scavenging (which depends on the La^3+^ ion) but will have a negligible effect on LaPO_4_ precipitation.

## Results

### PQQ-DHs in the ocean are primarily Ln dependent

We identified 6,328 unique PQQ-DH proteins in the microbial metagenomes (Fig. 1A), of which 61% contained the lanthanide binding motif, 12.5% contained the homologous calcium binding motif, and 26.5% contained a homologous sequence to the Ln and Ca binding domains but with neither the diagnostic Ln or Ca binding residues. The cofactor binding regions in most of these proteins is relatively narrow, so that Ln-dependence is usually observed at the exclusion of Ca-dependence (however see discussion of GDH). Sequences were manually classified based on their clustering with reference sequences and annotated following the previously proposed classifications from Keltjens *et al* [13]*,* resulting in 17 PQQ-DH families. In 14 of these families, most or all of the proteins contained the lanthanide binding motif (Fig. 1C andS2C). 2,424 reference sequences were used to construct the protein tree: 2,391 are annotated computationally but not experimentally characterized [13, 26, 27, 40, 69], while 30 correspond to experimentally characterized PQQ-DH enzymes (compilation provided in Table S1).

We explored the bacterial and archaeal metagenome assembled genomes (BacArcMAGs; MAGs) dataset [38], which is based on TARA oceans metagenomes from the SO and DCM. We found that 14 / 33 phyla and 20% of all 1,888 MAGs contained Ln-PQQ-DHs. Three *Acidobacteria* genomes and a group of fifteen *Pseudomonadales* UBA9145 genomes contained an unusually large repertoire of Ln-PQQ-DHs, with genes for up to 70 unique Ln-utilising enzymes in a single genome (Fig. 2A).

Spatial variation in the relative abundance of PQQ-DH genes in the metagenomes is modest (Fig. 3A,C,E, and G). However, community level transcript abundance (a proxy for expression) is highly spatially variable, falling into 3 distinct regimes: 1. A high temperature surface ocean regime dominated by the MDH XoxF5; 2. A low temperature surface ocean regime dominated by the MDH XoxF4 and ADH type 3; and 3. A deep water regime, dominated by ADHs types 6a, 2a, and 2b (Fig. 3B,D,F, and H). The samples from the deep chlorophyll maximum occupy the transitional zone between these regimes. Ln-dependent metabolism in the surface ocean is dominantly methylotrophic, whereas oxidation of multi-carbon alcohols (ethanol, propanol or butanol) dominates at depth. A higher fraction of unclassified sequences whose substrates are unknown are highly transcribed at depth.

Ca concentrations in seawater are around 10^−2^ M [70] while Ln concentrations are on the order of 10^−11^ M [71], yet the vast majority of microbial PQQ-DH enzymes in the ocean utilize Ln over Ca (Fig. 1C). This result, while surprising, aligns with an emerging picture that Ln-dependent PQQ alcohol DHs (PQQ-ADHs) are more diverse than their Ca-dependent counterparts in some previously explored environments [13, 25–28, 72], but expands this view to a far greater diversity of protein families, and a more nuanced picture involving metal cofactor heterogeneity within families previously thought to bind exclusively Ca (e.g., ADH type 4) or Ln (e.g., ADH type 2b). The dominance of Ln- over Ca-dependent PQQ-DHs in the global ocean is consistent with the experimentally demonstrated superior efficiency and preferential use of Ln- over Ca-dependent ADHs in the laboratory [17, 73].

### Putative Ln-dependent glucose and sorbose dehydrogenases

Based on our interpretation of the topology of the protein tree, glucose DHs (GDHs) and sorbose DHs (SDHs) respectively represent 15% and 8% of all PQQ-DHs in the metagenomes (Fig. 1B). The vast majority of putative GDHs and putative SDHs contain the Ln-binding motif (Fig. 1C), making them the most diverse groups of Ln-PQQ-DHs in the dataset. Among biochemically characterized (Ca-dependent) enzymes, SDHs exhibit relatively high levels of substrate promiscuity, while GDHs do not (Fig. S4 and Table S1). To our knowledge this is the first time that putative Ln-dependent GDHs and SDHs have been identified, expanding the possible role of Lanthanides in metabolism beyond volatile alcohols and aldehydes.

All Tara sequences in the clade designated as putative GDH contain the diagnostic “D-x-D” motif. We modeled the structure of GDH using AlphaFold2, which positions the two aspartate residues required for lanthanide binding with high confidence (pLDDT) and they are coordinated within interaction distance of the active site, reflecting Ln-binding (Fig. 1I and J). Conservation of the Ln-binding residues in the PQQ and substrate-binding active site across all Tara GDH sequences (Fig. 1C, S2C) indicates an important role in the enzyme's function. Within this clade we observe a tight clustering between several reference sequences including one biochemically characterized membrane-bound GDH (m-GDH, UniProt ID: P15877; labeled PQQ GDH group 1a, Fig. 1A and Fig. S2). The majority of the sequences in this group also contain transmembrane domains. These observations strongly support our interpretation that the proteins nested within the GDH 1a grouping are GDH in function. Putative GDH group 1b and group 2 do not contain transmembrane domains and are each supported by a single biochemically unconfirmed GDH reference sequence, so are less strongly supported (Fig. S2 B).

In m-GDH, the number of residues involved in metal coordination in the active site is unusually large compared with other PQQ-DHs, and these proteins can complex with a variety of metals such as Ca, Mg, Zn, and Sr [74]. Despite much published work on GDH, its native tertiary structure has not been resolved. The active site of m-GDH has previously been modeled based on calcium-dependent MxaF [75] and to our knowledge remains the only prediction of its structure. MxaF lacks the aspartate residue required for lanthanide binding [36] and therefore so does the most referenced model of m-GDH’s active site. However, in our analysis, the most similar protein structure to m-GDH (P15877) is Ln-dep PedH (E-value = 3.35e^−58^, PDB: 6ZCW) with a RMSD of 1.064 (between 327 pruned atom pairs, 47% of the amino acid sequence) which shares the two aspartate residues essential for Ln-binding. The biochemically characterized m-GDH and most of the computationally characterized GDH reference sequences do contain the lanthanide binding motif. Therefore, we predict that m-GDH is facultatively Ln-dependent and is promiscuous in its metal cofactor.

Two clades of SDH were resolved with strong support from reference sequences. These two clades share a common ancestor with a third clade (all of which are distinct at 33% similarity), which is composed of sequences exclusively containing the diagnostic “D-x-D” lanthanide-binding motif. The AlphaFold2 model of SDH is most similar in structure to the only SDH deposited on PDB, a calcium dependent sorbose dehydrogenase (PDB ID: 4CVB; E-value = 1.74e^−34^; RMSD of 1.039, between 454 pruned atom pairs, 83% of the amino acid sequence). The two diagnostic aspartate residues are coordinated with high confidence in the active site, and within interaction distance, reflecting Ln-binding (Fig. 1L and M).

Many different putative Ln-PQQ sorbose DHs occur in the genomes of a relatively small number of organisms that possess these genes (mostly Acidobacteria and Gammaproteobacteria) (Fig. 2A). By contrast, putative Ln-PQQ-GDHs occur in low numbers in each genome, and the organisms in which they occur are phylogenetically diverse (Fig. 2A) and are geographically widespread (Fig. 3A–F). Yet expression levels of GDHs and SDHs in the surface ocean are low, with just 1% each of total PQQ-DH transcript abundance (Fig. 3H).

## Ln-dependent methanol dehydrogenase genes are low abundance but highly transcribed

Methanol dehydrogenase (MDH) genes are low abundance compared with other PQQ-DHs in the metagenomes but dominate community PQQ-DH transcript abundance, particularly in the surface ocean. Two clades, *xoxF5* (n = 63) and *xoxF4* (n = 11), which are the dominant PQQ-MDH clades reported in a variety of environments [15, 26, 76], together represent just 1% of all unique PQQ-DH genes and 1% of the gene abundance, but 55% of transcript abundance (Fig. 3G and H). In the surface ocean, *xoxF4* genes are more highly expressed in the high latitudes while *xoxF5* genes are more highly expressed in the low and mid latitudes (Fig. 3B). We note that in *M. extorquens* AM1, apo-XoxF is expressed in the absence of lanthanides and methanol and is thought to be a mechanism for Ln detection and MxaF regulation [19]. However, we did not identify any genomes with co-occurring *mxaF* and *xoxF* (Fig. 2A).

We found that Ln and PQQ-dependent dehydrogenases are the most abundant, and by far the most highly expressed, methanol oxidation genes in the ocean metagenomes. Of prokaryotes in the <3 μm fraction (the >3 μm fractions are not included in OM-RGCv2), Ln-dependent MDHs comprise 80% of gene abundance and 99% of transcript abundance across all putative methanol oxidation genes in the global ocean database, including genes encoding non-Ln dependent PQQ-DHs, NAD-dependent DHs, and eukaryotic O_2_-dependent methanol oxidases (Fig. 4A and  S5). The availability of Ln therefore appears to be central to methanol oxidation in the ocean.

We found that XoxF4 enzymes are exclusive to the family *Methylophilaceae* of Gammaproteobacteria whereas XoxF5 enzymes are widespread across Proteobacteria, consistent with previous findings [15]. Some strains of *Methylophilaceae* (LD28 and PRD01a001B) are suggested to be psychrophilic [77], which could explain our observation that temperature is the environmental parameter most highly correlated with the separation of *xoxF4* and *xoxF5* expression (Fig. 3H). To identify the dominant Ln-utilising organisms we created a tree of the most highly expressed *xoxF* genes from the metatranscriptomes with all *xoxF* genes identified in the MAGs (Fig. 4B, S6, and S7). The three most highly expressed *xoxF5* sequences group with *xoxF5* sequences derived from three alphaproteobacterial genomes previously classified as *Rickettsiales* [38]. Further phylogenetic characterization placed these genomes in a clade with several other MAGs from the Tara oceans database also previously classified as *Rickettsiales* (Clade X, Fig. 4B, S6 and S7; and an orange star in Fig. 2 for wider phylogenetic context). Clade X is resolved with high confidence, but the phylogenetic relationships between Clade X and its neighboring groups including *Roseobacter*, SAR116*, Rickettsiales* [62], and the *Pelagibacterales* (previously SAR11) were resolved with low confidence. The nature and mode of life of this apparently central group requires further investigation.

We explored the possible sources of methanol to methanol-oxidizers. Ln-PQQ-MDHs are employed by some methanotrophic communities [78, 79], however, an analysis of methanotrophy marker genes (pMMO and sMMO) in the metagenomes and MAGs did not reveal any genes associated with methane oxidation, only ethane and hydrocarbon monooxygenases. The substrate for the Ln-PQQ-MDHs in the analyzed samples is therefore likely not methane-derived methanol (Fig. S8 and S9). Pectin, the primary source of methanol in the phyllosphere, which is the habitat of many well studied methylotrophs, is also in short supply in the open ocean. Single celled algae, which have been shown to produce methanol in significant amounts [80], are a possible source of methanol in the open ocean.

### Identification of REE transporters and putative lanthanophores

Several mechanisms involving manipulation of the chemical environment have been proposed to account for lanthanide solubilization [81, 82], but which are not feasible in seawater. The hypothesized existence of lanthanophores, Ln chelators analogous to siderophores for iron [73], has been invoked to explain Ln utilisation in aqueous environments where low Ln solubility might limit bioavailability. Metallophores produced by biosynthetic gene clusters (BGC) with TBDT are known to play an important role in metal uptake in several species of bacteria [83, 84]. The mechanisms required for lanthanide acquisition and transport are still relatively unknown but are expected to be analogous to siderophore-mediated iron transport [85, 86]. The alphaproteobacterium *M. extorquens* AM1 has been shown to biosynthesize methylolanthanin when grown with poorly soluble Nd_2_O_3_, [35]. We analyzed over 14,000 TBDTs from 1,888 MAGs and identified 41 TBDTs from 11 orders across Alphaproteobacteria and Gammaproteobacteria that clustered with four experimentally confirmed TBDTs from *Methylobacteriaceae* (Fig. S3)*.* Due to the poor solubility and scarcity of Lns in the environment they are expected to be actively taken up via TBDTs. Despite a large number of diverse Gram-negative bacteria with Ln metabolisms, identifying their REE TBDTs is limited by the small number of REE TBDTs and their lack of full biocharacterisation e.g. identification of the REE-ligand binding residues, to use as a signature to find more divergent REE TBDTs outside of those with similarity to *Methylobacteriaceae* REE TBDTs. We also found 19 genomes containing Ln-PQQ-DHs that also contained a nonribosomal peptide synthetase BGC that contained the TBDT (Fig. 2A, red stars). Two of these genomes also contained over 30 lanthanide dependent enzymes, while four genomes additionally contained FecCD transmembrane protein (Type II ABC importer) and Peripla_BP_2 (substrate binding domain) in the BGC. The presence of these proteins has been shown to positively correlate with metallophore uptake [53]. The biosynthetic gene clusters for three of these putative metallophores (Fig. 2A, purple stars, B and D, numbered 4, 17, and 19) contain TBDTs that cluster with methylolanthanin’s TBDT (*mluA,* META1p4129) (Fig. 2B) suggesting that they may transport methylolanthanin. These also cluster with known iron transporters PiuD, BauA, FhuA, FoxA, FptA, and FhuE. This is consistent with methylolanthanin TBDT activity which also responds to iron limitation indicating a dual role involving lanthanide and iron sequestration [35]. All three metallophores also contained petrobactin-like biosynthesis and acetyltransferase genes, consistent with methylolanthanin. The *Thalassospira* MAG (TARA_IOS_50_MAG_00003) from the Alphaproteobacteria which contains metallophore 4 and the *Salinisphaera* MAG (TARA_PSW_86_MAG_00118) from the Gammaproteobacteria which contains metallophore 17 both contained Ln-PQQ-DHs, providing independent, compelling evidence of lanthanide utilisation. On the balance of this evidence, we suggest that metallophores 4, 17, and possibly also 19 are likely capable of complexing lanthanides.

### Marine biogeochemistry of the lanthanides

We explored the environmental factors controlling the abundance of lanthanide utilising enzymes. We define fLn_G_ as the fraction of PQQ-DH genes present in each metagenome that contain the Ln-binding motif (Fig. 5A and  5B). We define a similar ratio, fLn_T_ for transcripts in each metatranscriptome (Fig. 5C and  5D). By normalizing the abundance of Ln-utilising enzymes to that of functionally similar enzymes that use a different metal cofactor, we are aiming to ask when Ln utilising enzymes are favored over Ca- or other metal- dependent alternatives, and to minimize bias that may result from differences in the metabolic role of these enzymes. Broadly, we found that fLn_G_ is highest in the more oligotrophic regions of the ocean, and lowest in the high-nutrient low-chlorophyll (HNLC) regions of the ocean. High values of fLn_T_ are found everywhere, but low values are only found in the HNLC regions. HNLC regions of the ocean are characterized by high phosphate concentrations, which has been suggested to place an upper limit on the solubility of Ln [87]. We suggest that the occurrence of low values of fLn_G_ and fLn_T_ exclusively in regions of high phosphate concentration reflects the extremely low Ln concentrations in these regions (Fig. 6). Inputs of Ln to the surface ocean are highly spatially heterogeneous including terrigenous input from rivers [88], wind-blown dust, subaqueous volcanism and upwelling of nutrient-rich deep water [89]. Outputs of Ln from the surface ocean include: 1. Biological uptake; 2. Scavenging via adsorption to sinking particles including a) phytoplankton biomass [90], and b) clay minerals [91]; and 3. Precipitation as LnPO_4_.

Outputs 1 and 2a are likely most important in regions where production and export of organic matter is high. However, these effects may be lessened by the replenishment of Ln to the surface ocean that accompanies the replenishment of whichever nutrient limits phytoplankton growth rate (e.g. wind-born Fe and Ln from dust in the Southern Ocean [92, 93]). The efficiency of adsorption reactions (outputs 2a and 2b) depends on the concentration of the free Ln^3+^ ion, which increases with decreasing pH, as a higher fraction of total Ln is in the form Ln^3+^ due to the decreased abundance of the dominant inorganic ligand, CO_3_^2−^ (Fig. 6B). LnPO_4_ is a highly insoluble salt (K_sp_ ~ 10^−26^ - 10^−25^ [94]). Rates of LnPO_4_ precipitation are greater in waters that are more oversaturated with respect to LnPO_4_. Output 3, therefore, depends on the solubility product [Ln^3+^][PO_4_^3−^]. The concentration of the PO_4_^3−^ ion increases with pH and total phosphate, while the free Ln^3+^ ion is a smaller fraction of total Ln at high pH [95]. The opposing effects of pH on Ln^3+^ and PO_4_^3−^ result in a subtle optimum relationship, with lowest concentrations of total Ln at LnPO_4_ saturation occurring at around pH 8.1, and modest increases at lower or higher pH (Fig. 6C). The value of [Ln^3+^][PO_4_^3−^] is most strongly determined by the concentration of total phosphate (Fig. 6C). This is supported by the strong negative relationships between total phosphate and fLn_G_ and fLn_T_ (Fig. 5B and  5D).

We conclude that Ln scavenging efficiency is likely most strongly controlled by pH (higher at low pH), while LaPO_4_ precipitation is most strongly controlled by phosphate (higher at high total phosphate). In the ocean, phosphate concentrations and pH negatively covary, so these removal fluxes cannot currently be decoupled. However, both processes will be more efficient where phosphate is high and pH is low such as the high-latitude HNLC regions, and less efficient where PO_4_ is low and pH is high such as the ocean gyres (Fig. 5 and 6). Even accounting for these processes, there is a large spread in both fLn_G_ and fLn_T_, the simplest explanation for which is that inputs of Ln are temporally and spatially variable. At high pH and low phosphate, there is negligible depletion of surface ocean Ln, which is always available at sufficient concentrations (fLn_T_ is always high), while at low pH and high phosphate, the steady state concentration depends on the balance between inputs and outputs (fLn_T_ can be high and low).

## Discussion

### Evolutionary trade-offs between Ln- and Ca- dependent DHs

Our results reveal that Ln-dependent forms of PQQ-DHs dominate over the canonical Ca-dependent forms, despite the billion-fold higher concentrations of Ca over Ln in seawater. Ln are superior Lewis acids to Ca, and have been predicted to be more effective activators of the redox cofactor PQQ [96]. This contrast between Ln and Ca in enzyme efficiency and metal availability in natural environments highlights an evolutionary trade-off which our results show strongly favors quality over quantity of the available metal cofactor. Only in those regions of the ocean where stable Ln concentrations are practically zero (i.e. HNLC regions where phosphate concentrations are extremely high) do we find communities where Ca-dependent forms are favored.

Although expression is dominated by MDH in the surface ocean and by ADHs at depth, our results also suggest that the lanthanides have metabolic functions beyond a role in the oxidation of volatile alcohols and aldehydes. The diverse families of Ln-dependent putative monosaccharide dehydrogenases, particularly GDH, are phylogenetically widespread (Fig. 2A), but are not highly expressed (Fig. 3B, D, F, H). These monosaccharide dehydrogenases may play an important metabolic role in the modern ocean, but only in certain circumstances not captured in the data. Ln concentrations in Archean seawater when bacteria evolved were likely much higher than today, owing to low pH and low phosphate concentrations (which may have been very low [97, 98], however this remains hotly debated [99, 100]), and would have decreased significantly via scavenging by iron oxides during the oxygenation of the ocean. A deep evolutionary origin for Ln-dependent MDH has been proposed previously [13]. Our results support this conclusion and further suggest that bacterial Ln-dependent enzymes might have been more important during the early evolution of aerobic respiration than today.

### Climatic implications of a coupling between Ln and C cycles

We showed that methanol dehydrogenases are the most highly expressed Ln-dependent enzymes in the surface ocean. While we hypothesize that the source of methanol is planktonic algae, methanol cycling in the surface ocean is poorly understood. The absence of methane marker genes in the samples represented in the dataset does not preclude the likely importance of methane-derived methanol in regions not captured in this dataset. The availability of Ln in seawater is a function of pH, and thus is sensitive to changing seawater CO_2_ concentrations associated with Anthropogenic emissions. If Ln-dependent methane oxidation is a significant process in the modern ocean, it would constitute a sink of a highly potent greenhouse gas. This sink would be pH dependent, and thus an important biogeochemical climate feedback that should be investigated. Future work should therefore target regions of methane influx to the ocean such as shelf regions where massive amounts of methane are released from subsurface deposits, and submarine volcanism.

The coupling between the marine Ln and carbon cycles likely has implications for Earth’s climate (Fig. 6). These interactions may drive climate feedbacks, for example, in Anthropogenic future oceans, the efficiency of Ln scavenging will likely increase following decreases in seawater pH. On longer timescales oceanic phosphate concentrations are projected to increase on millennial timescales as a result of increased weathering [101], which would likely cause an increase in LnPO_4_ precipitation. Both effects would drive a decrease in the surface ocean concentration of Ln, and therefore a reduction in the rate of Ln-dependent alcohol oxidation to CO_2_, manifesting as a negative feedback to increasing atmospheric pCO_2_.

### Implications for understanding biogeochemical Ln patterns

Lanthanide concentrations in seawater exhibit nutrient-like vertical profiles, being low in surface waters, and increasing, and ultimately plateauing, with depth [30]. Such profiles are typically reflective of biological uptake and remineralization at depth. The concentration profiles of Ln have been reconciled with their assumed lack of biological utility with various scavenging models. However, our findings demonstrate that Ln is indeed a nutrient, and therefore that such behavior should be expected.

Usually referred to as the REEs in the field of ocean geochemistry, the relative concentrations of Lns in seawater and in sediments have been extensively used to explore ocean circulation and hydrothermalism in the modern ocean and over geologic time [102]. With the exception of Ce, which is oxygen sensitive, differences in the efficiency of removal fluxes between the Lns are attributed to relative stability of aqueous complexes [95]. Biological utilisation of Ln in seawater has, until very recently, been assumed to be unimportant [31]. The deepwater horizon disaster in the Gulf of Mexico provided the first circumstantial evidence of biological Ln utilisation. During this event, a rapid increase in water column methane concentrations drove a bloom in water column methanotrophy that was coincident with a dramatic decrease in the lightest Ln in seawater [24]. Our results show that biological Ln utilisation is not restricted to discrete events or to methanotrophy, but is ubiquitous and diverse. Microbial uptake and export thus likely imparts a previously unaccounted for fractionation of the Lns, with more efficient uptake of the light rare earths over the heavy rare earths which must be constrained and factored into future geochemical analyses.

### Potential industrial applications

Despite their significant economic value, efficient extraction and purification of Ln from raw materials remains an unsolved challenge [103]. The methylolanthanin-bearing organisms that we highlight will be appealing targets for use in bioleaching and biomining efforts. Biological utilisation also discriminates heavily between Ln. Observed bacterial utilisation of lanthanides has thus far only involved La to Gd, and recently Pm [104] (even though it does not exist in nature), with a preference for the lightest Ln of the series, La, Ce, and Nd. Small periplasmic lanthanide binding proteins such as landiscerin, meanwhile, have shown a preference for heavier lanthanides, acting as a siphon leading to potential export of heavier lanthanides [22]. Despite being the weaker Lewis acids, and conflicting theoretical predictions of whether Ln-MDH preferentially binds the lighter [105] or heavier [106] of the series, preferential use of these light Lns is clear and may have evolved owing to their higher abundances in seawater. This discrimination is likely to occur in the periplasm [107] although the underlying mechanisms are in the early stages of characterisation. The separation of lanthanides from one another remains a significant technological challenge for industry, but one that microbes appear to have already solved.

### Limitations of the study

The Tara oceans dataset is global in its extent, but not comprehensively so. Much of the Southern Ocean, and the west Pacific Ocean are not represented, and ship-based sample collection necessarily represents a discrete time interval from within an annual cycle. The metagenomics and metatranscriptomics results are based on samples from the <3 μm fraction, which isolates the free-living prokaryotic component of the ocean microbiome, and therefore does not include the gut microbiomes of zooplankton, although these are included in the MAGs dataset. The scope of this work is so huge that it can only be addressed via computational methods that leverage high throughput sequencing and bioinformatics tools. Evidence to date supports the Ln binding motif being sufficient and necessary for Ln binding, but this will need to be verified for unknown enzymes. An important outcome of this work is the identification of targets for future research. Specifically, biochemical studies will be required to corroborate the Ln-dependence and the substrate specificity of the putative GDHs and SDHs described here, and the specificity and chelation efficiency of the putative lanthanide metallophores.

## Conclusions

Only 12 years ago the lanthanides were thought to have no role in biology, yet enzymes that depend on them are found in a fifth of all microbial genomes in the surface ocean. The dominance of the marginally more efficient Ln-PQQ-dependent enzymes over their Ca-dependent equivalents, despite the vastly higher abundance of Ca over Ln in seawater, suggests that efficiency confers a far greater advantage than the availability of their metal cofactor. Ln-dependent metabolism is an unappreciated component of the ocean microbiome that plays a central, yet almost entirely overlooked, role in marine carbon biogeochemistry. Uncovering the details of these biogeochemical feedbacks and implications for climatic regulation is an important aim for future research.

## Supplementary Material

Tara_for_ISME_SI_V4_wraf057

Fig_S2A_wraf057

Fig_S2B_wraf057

Fig_S2C_wraf057

Fig_S2D_wraf057

Supplementary_Datas_wraf057

## Data Availability

The sequence datasets analysed during the current study are available in the Tara Oceans metagenomic and metatranscriptomic Ocean Microbial Reference Gene Catalog v2.0 (OM-RGCv2), bacterial and archaeal BacArcMAGs, and Marine atlas of tara ocean unigenes (MATOU). Link to all datasets: https://tara-oceans.mio.osupytheas.fr/ocean-gene-atlas/. The biogeochemical datasets used are the WOA2018 (https://www.ncei.noaa.gov/access/world-ocean-atlas-2018/) for phosphate concentrations and the CO2OceanSODA-ETHZ (https://www.research-collection.ethz.ch/handle/20.500.11850/474239?show=full) for surface ocean carbonate chemistry and pH.
