# Supplementary material for: Extensive and diverse lanthanide-dependent metabolism in the ocean
Source: ISME J. 2025 Apr 23;19(1):wraf057. doi: 10.1093/ismejo/wraf057 (PMC11996626; doi:10.1093/ismejo/wraf057)
Supplement: Tara_for_ISME_SI_V4_wraf057 [file tara_for_isme_si_v4_wraf057.pdf]

Supplementary Materials for

Extensive and diverse lanthanide-dependent metabolism in the ocean

Marcos Y. Voutsinos[1,2], Jillian F. Banfield[1,2,3,4,5] & Harry-Luke O. McClelland[1,6,*]

1. School of Geography, Earth and Atmospheric Sciences, The University of Melbourne, Melbourne, VIC, Australia
2. Department of Microbiology, Biomedicine Discovery Institute, Monash University, Melbourne, VIC, Australia
3. Department of Environmental Science, Policy and Management, University of California, Berkeley, CA, USA
4. Department of Earth and Planetary Science, University of California, Berkeley, CA, USA
5. Innovative Genomics Institute, University of California, Berkeley, CA, USA
6. Department of Structural and Molecular Biology, Darwin Building, University College London, London, UK

*Corresponding author. Email: h.mcclelland@ucl.ac.uk

The PDF file includes: Figures S1 to S9 and Table S1

Figures

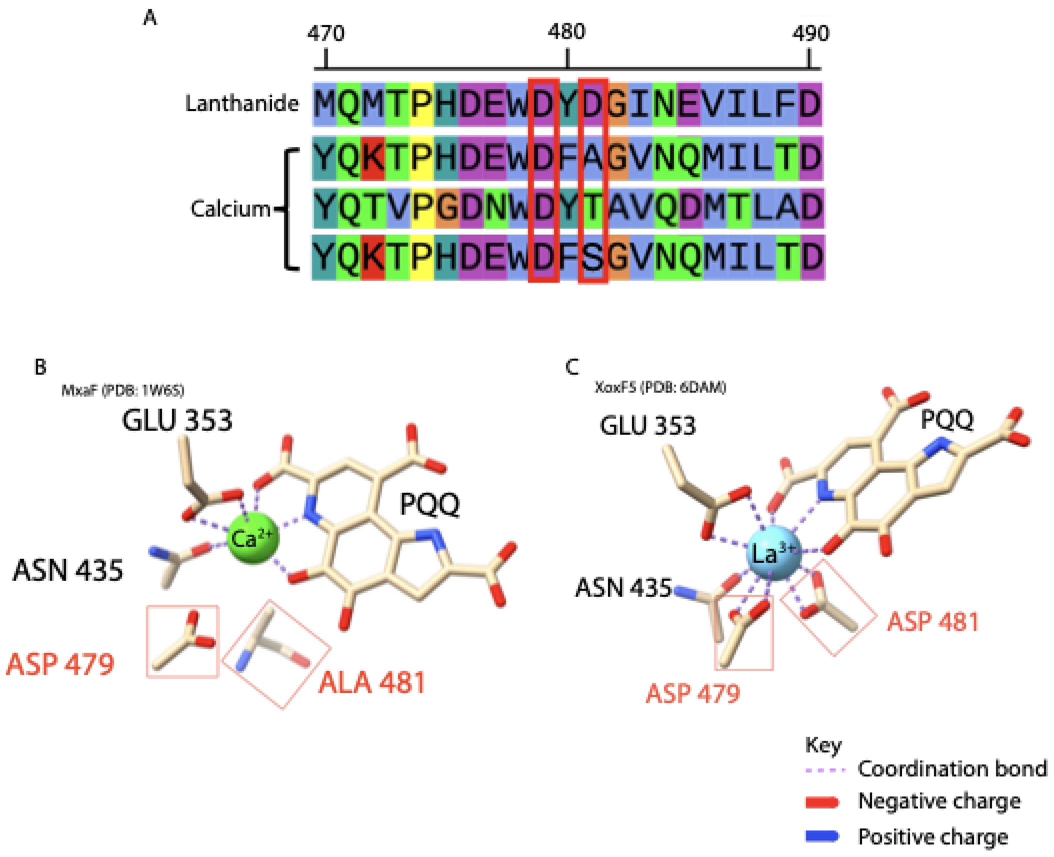

Figure S1: **(A)** Multiple alignment of the cofactor binding domain used to classify the metal dependence of pyrroloquinoline quinone dehydrogenase sequences identified in this study. Aspartate$_{479}$ (D) is required for catalytic activity, aspartate$_{481}$ is required for lanthanide binding, and alanine$_{481}$ (A), serine$_{481}$ (S), or threonine$_{481}$ (T) is diagnostic of calcium dependence. Unknown sequences classified based on the absence of aspartate, serine, threonine, or alanine at AA$_{481}$. **(B)** Active site of MxaF (PDB ID: 1W6S) illustrating the conserved aspartate$_{479}$ (ASP) and alanine$_{481}$ (ALA) residues (red boxes) conserved in Ca-dependent PQQ dehydrogenase enzymes [1]. **(C)** The active site of XoxF5 (PDB ID: 6DAM) illustrating the two ASP$_{479,481}$ residues (red boxes) that are required for lanthanide coordination.

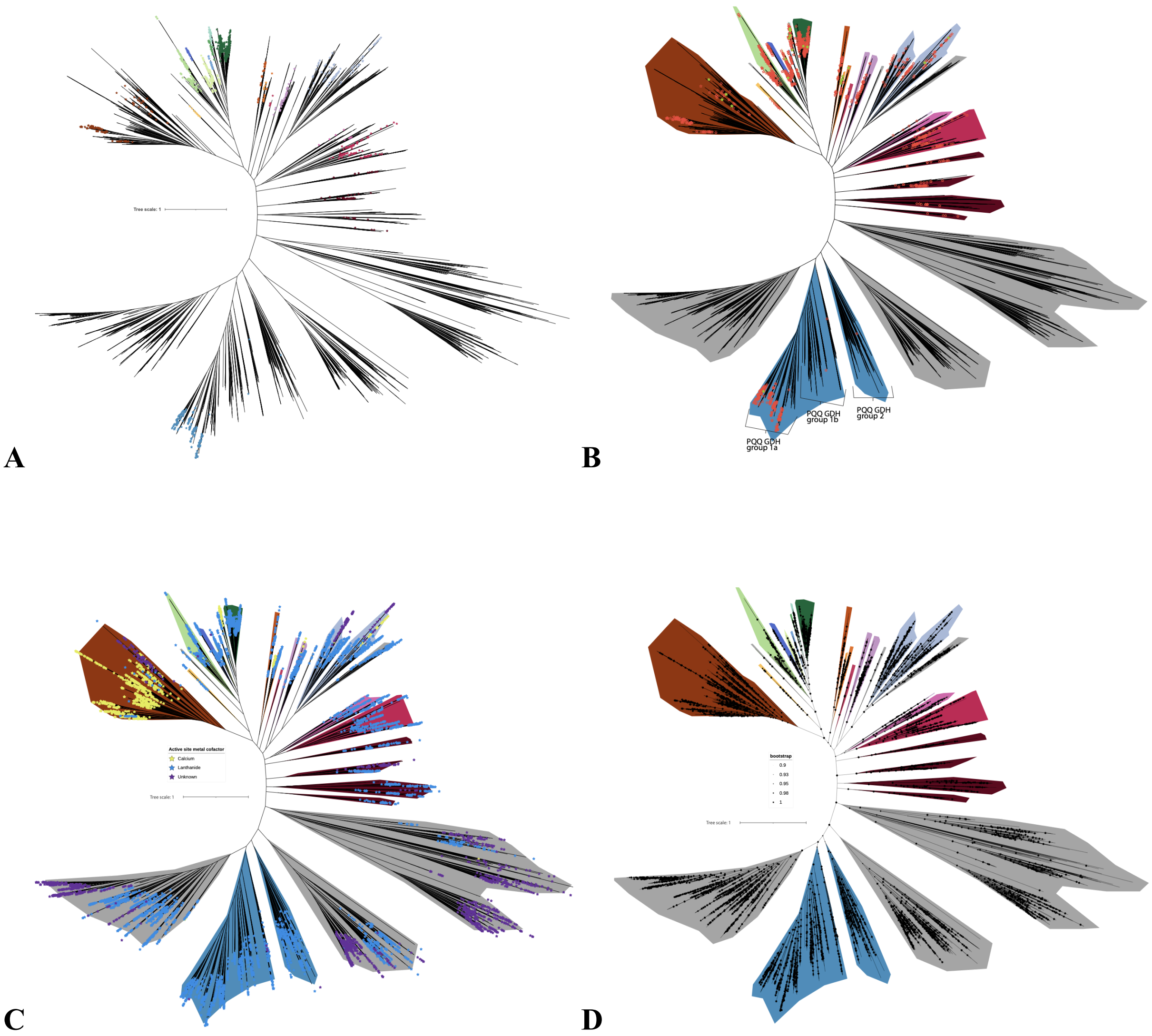

Figure S2: Approximately maximum-likelihood protein tree of 6,328 PQQ-DH sequences taken from this study and 2,424 PQQ-DH reference sequences taken from [1–5] (see figure 1C for color key). **(A)** Unshaded tree used to classify the 6,328 PQQ-DH sequences from this study. The stars represent reference sequences and are coloured based on their reported substrate activity. **(B)** Shaded tree representing our interpretation of Figure S2A. The solid green stars represent biochemically confirmed (primary sequences) proteins and hollow red stars represent previously classified sequences (secondary sequences) taken from the literature. The tree in Figure 1A in the main manuscript has this topology with the starred sequences removed. **(C)** The active site metal cofactors of each sequence are illustrated based on the presence of a lanthanide binding residue (blue), or, if absent, calcium (yellow) or an unknown metal cofactor (purple). **(D)** Bootstrapped phylogenetic tree with black dots at the nodes representing local support values > 90%. The tree reliability was calculated using Shimodaira-Hasegawa [6].

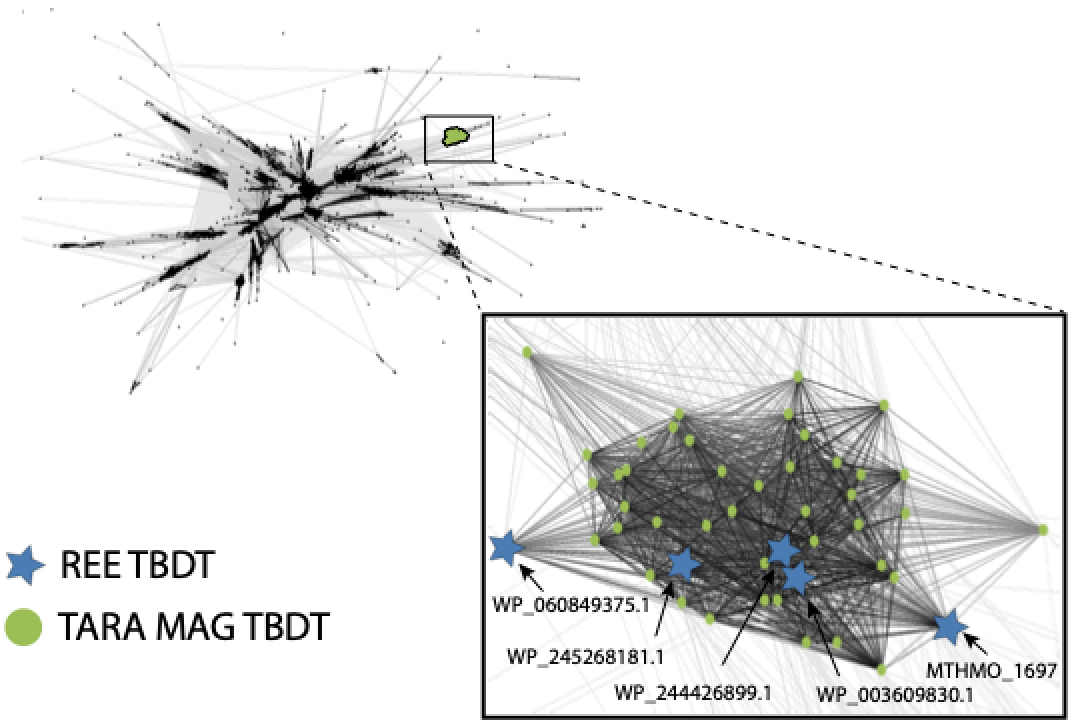

Figure S3: Sequence similarity network of more than 14,000 TonB dependent transport (TBDT) homologs derived from the 1,888 TARA MAGs with experimentally confirmed REE TBDT's reference sequences. Each point represents an individual protein, grey lines represent BLASTp E-values better than $1x10^{-10}$ with darker lines representing higher sequence similarity. The black box shows 41 TBDTs (green dots) from TARA MAGs clustering with REE TBDT (blue stars, labeled with protein accessions).

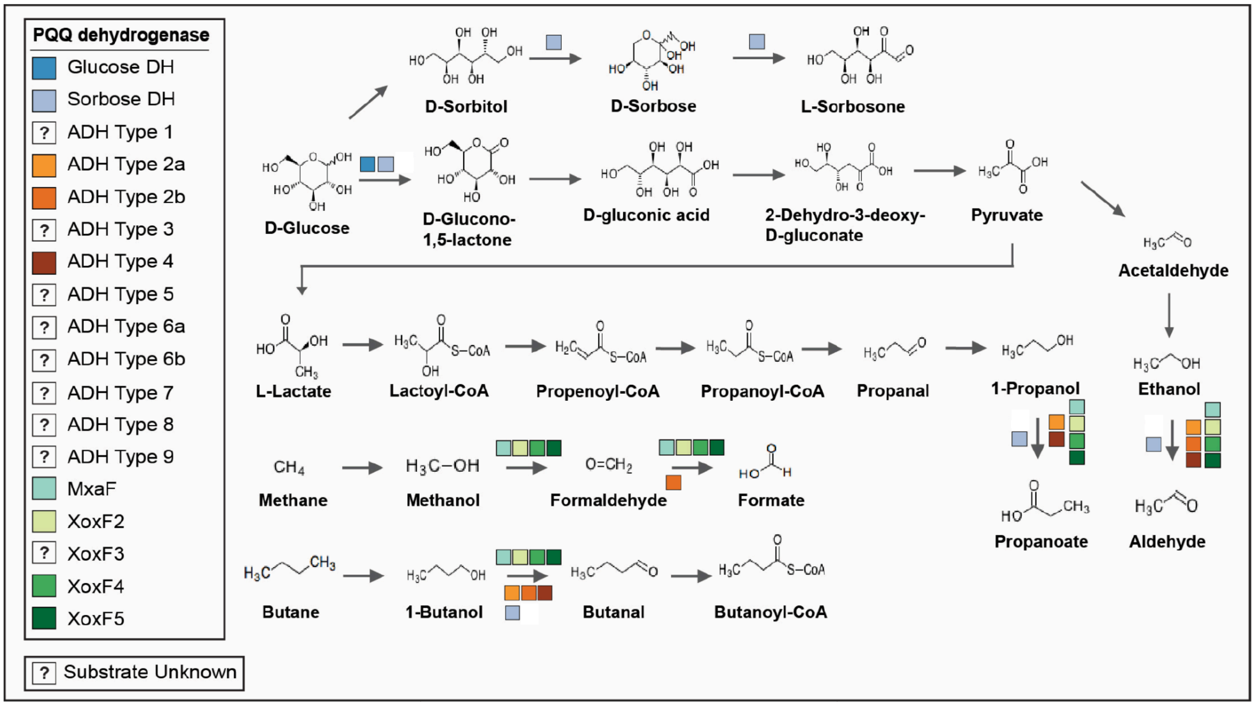

Figure S4: Summary of experimentally confirmed metabolic activities of pyrroloquinoline quinone dehydrogenases that were identified in this study. See Table S1 for references.

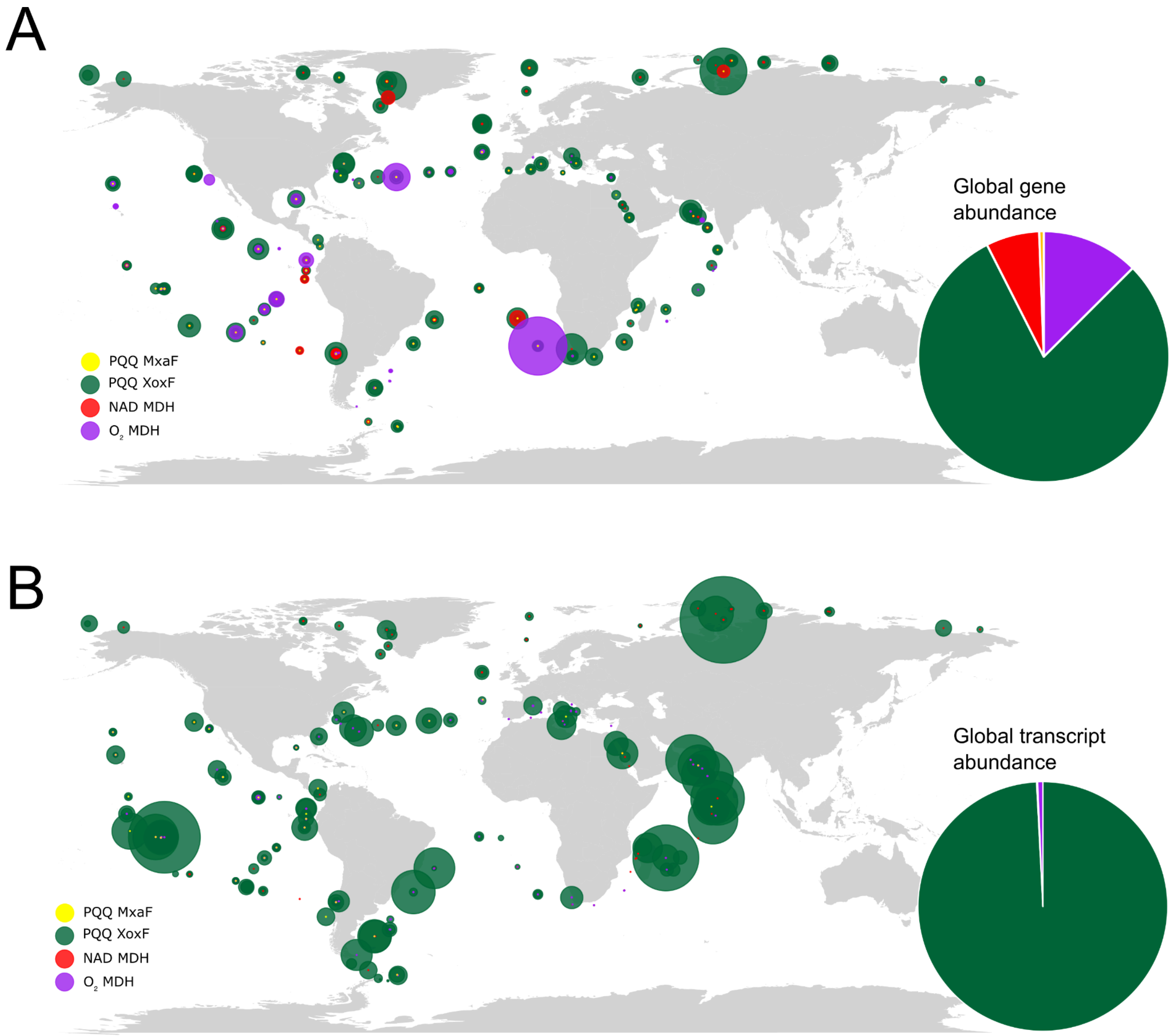

Figure S5: Maps of PQQ MxaF, PQQ XoxF, NAD MDH and O_2 M-oxidase in the global ocean **(A)** gene abundances and **(B)** transcript abundances in the combined surface ocean, deep chlorophyll maximum and mesopelagic. PQQ MxaF, PQQ XoxF and NAD MDH abundances were derived from the 0.22-3 μm size fraction and O_2 MDH abundances were derived from the 0.8-2000 μm size fractions.

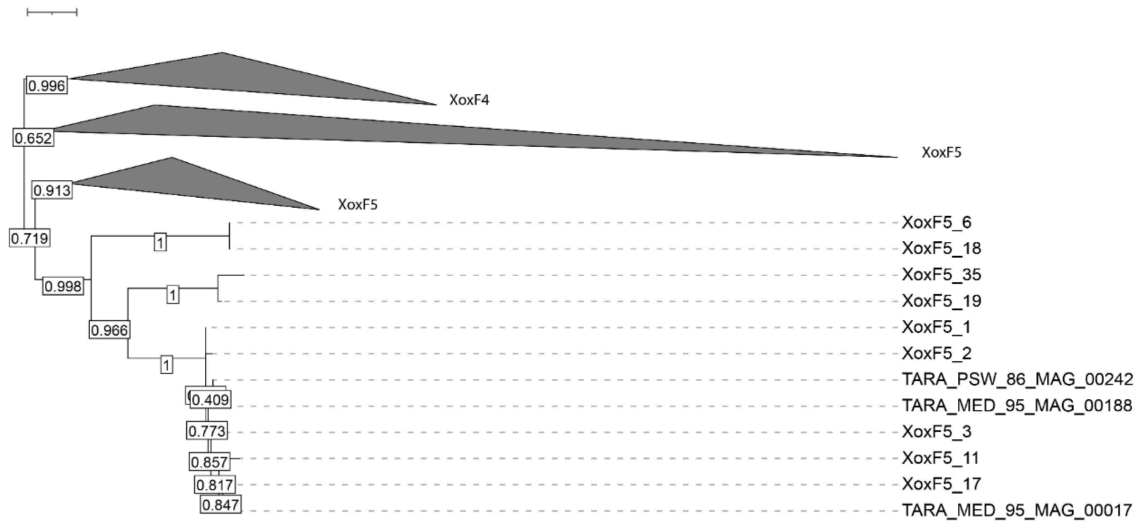

Figure S6: Phylogeny of the most highly expressed XoxF sequences from the metatranscriptomes and XoxF sequences from the MAGs. Metatranscriptomic XoxF5 sequence labels amended with abundance rank. Scale bar represents 0.1 substitution per site.

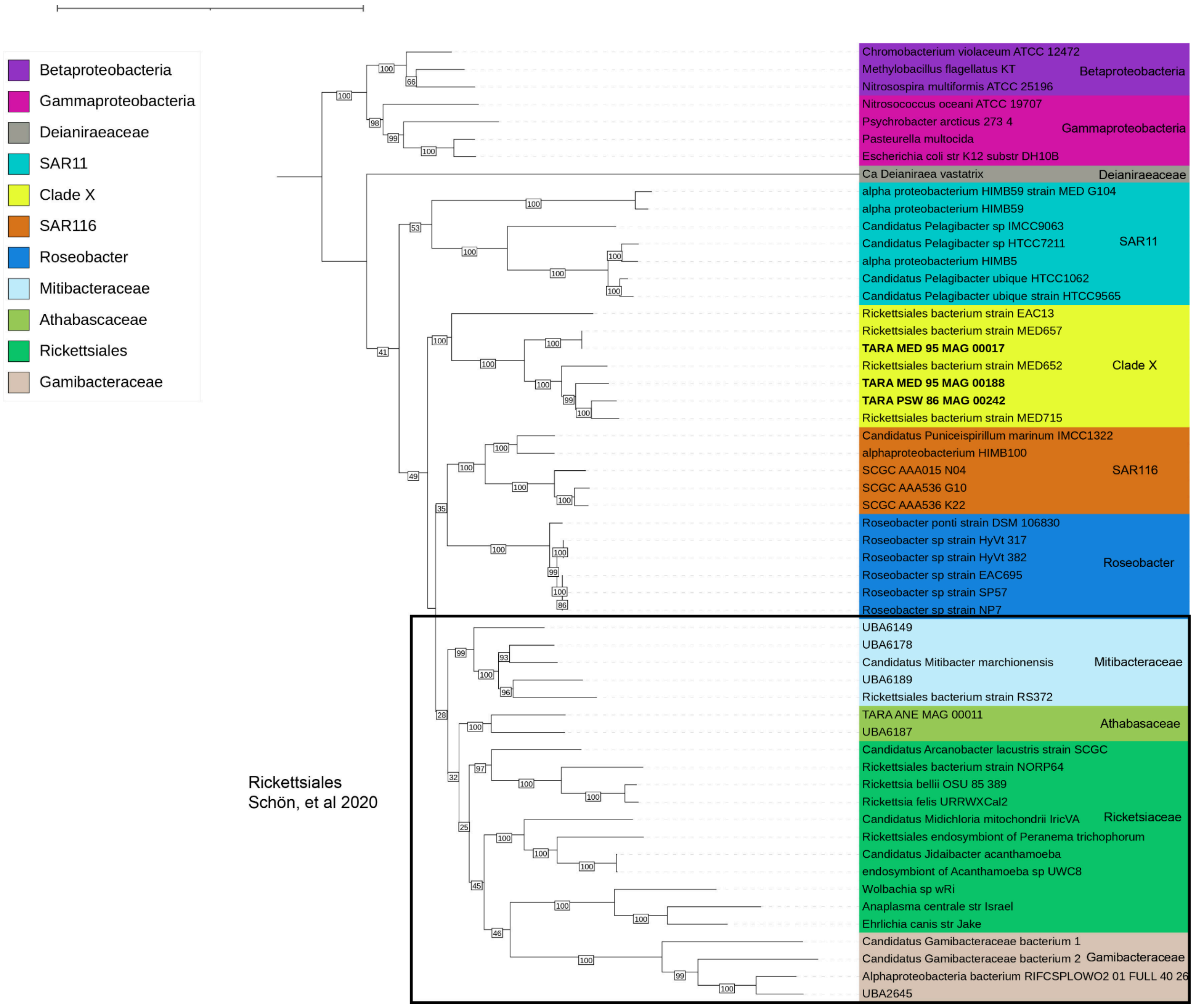

Figure S7: Expanded phylogenetic tree of the three Alphaproteobacteria genomes (Clade X) identified in Figure S6 to contain *xoxF5* sequences that group closely with the most highly expressed *xoxF5* sequences. The phylogenetic tree was constructed with a concatenated alignment of 16 ribosomal phylogenetic marker proteins (see methods). Reference genomes are taken from [7–9]. Scale bar represents 1 substitution per site.

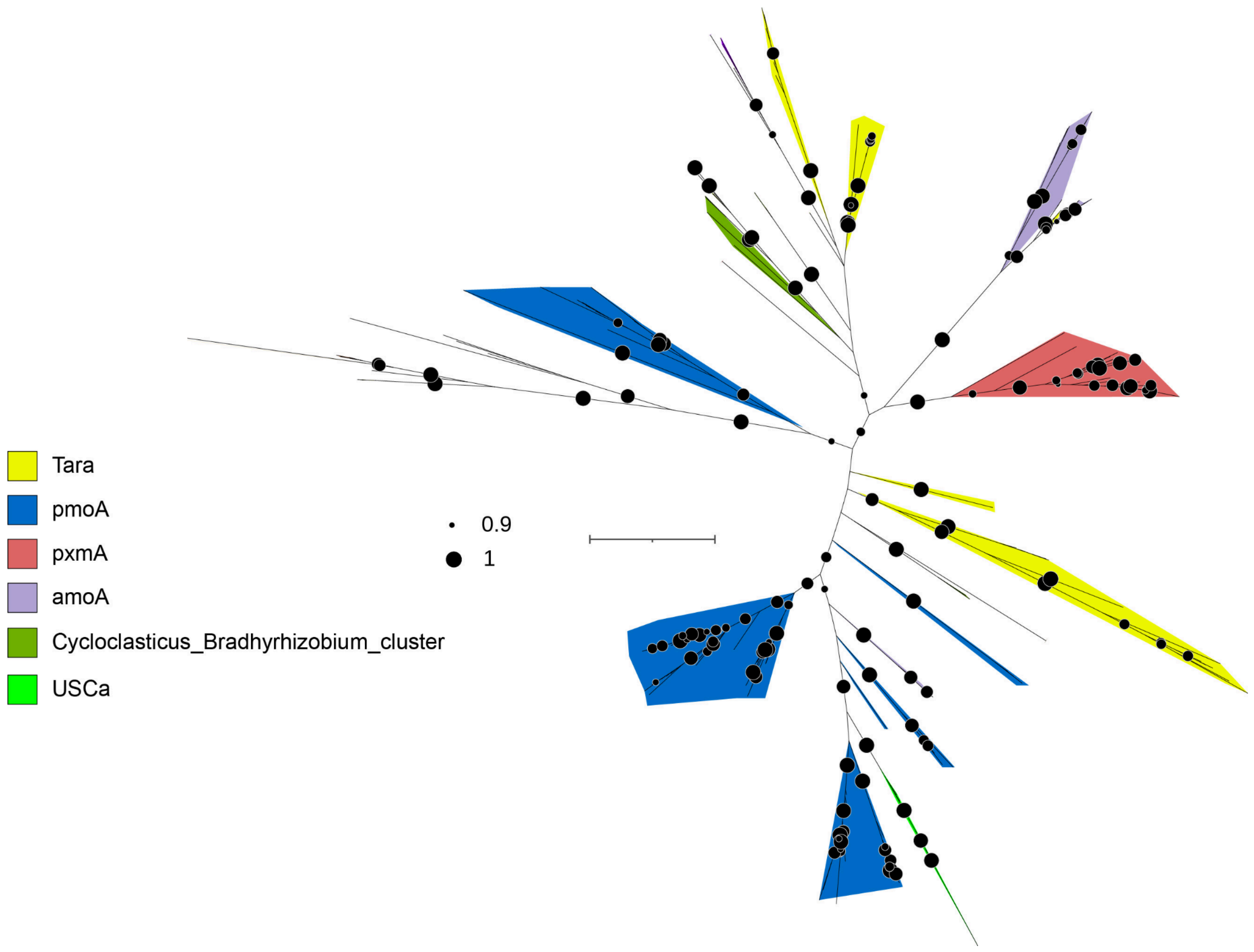

Figure S8: Subset of monooxygenase tree showing TARA monooxygenase homologs (yellow) derived from the TARA metagenomes and MAGs do not cluster with particulate methane monooxygenase (pmoA, blue) sequences. Tree is a subset of Supplementary data S2 with 27 highly divergent tara sequences pruned from the tree for better visualization. Reference sequences taken from Singleton et al. [10]. Bootstrap values greater than 0.9 are represented by black circles. Scale bar represents 1 substitution per site.

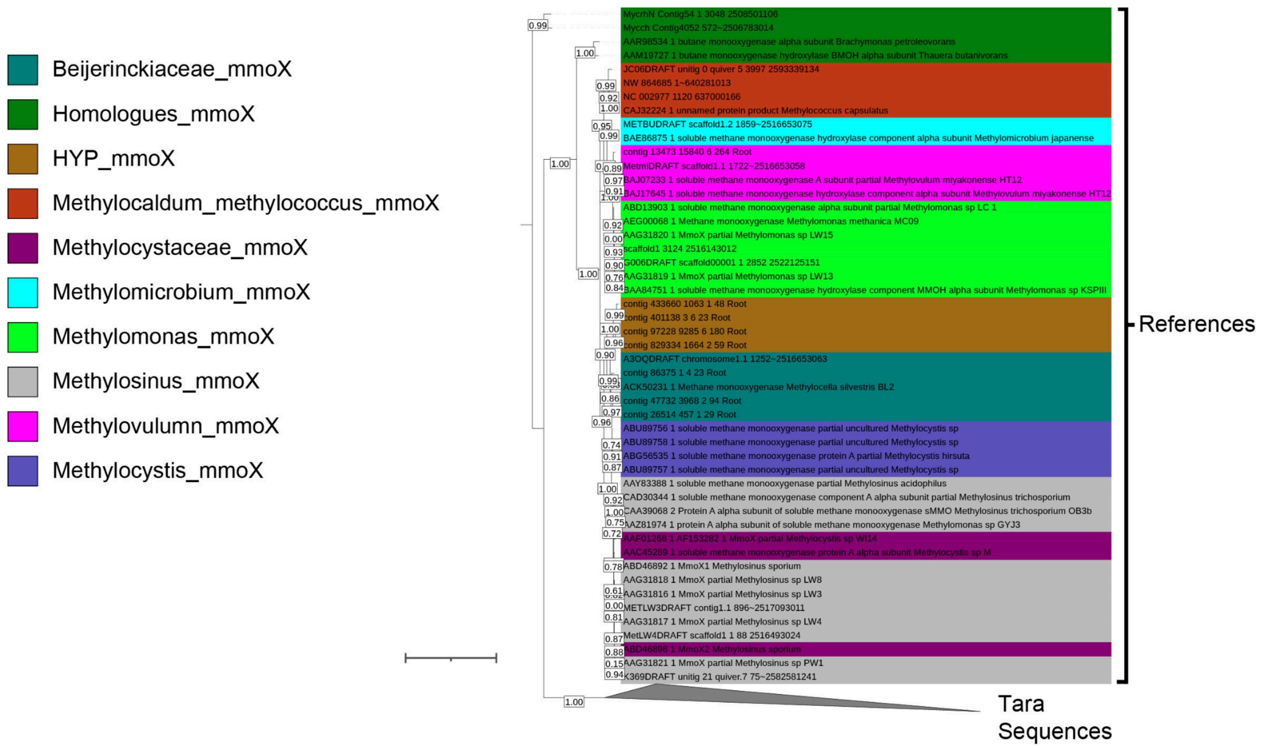

Figure S9 : Phylogeny of homologous soluble methane monooxygenase, MmoX proteins recovered from the TARA metagenomes and MAGs. None of the TARA sequences analyzed cluster within the reference sequences from Singleton et al. [10]. Scale bar represents 1 substitution per site.

Table S1. Compilation of biochemically confirmed PQQ dehydrogenase proteins. Reference sequences used in this study. Identifiers marked with an asterisk are PDB IDs.

Bacteria	Substrate	Identifier	Classification	Active site metal cofactor	Literature
Comamonas testosteroni	alcohol	*1KB0	ADH Type 4	calcium	[11]
Pseudomonas putida HK5	ethanol	*1KV9	ADH Type 4	calcium	[12]
Pseudomonas putida HK5	ethanol	*1YIQ	ADH Type 4	calcium	[13]
Pseudomonas aeruginosa	ethanol	*1FLG	ExaA (ADH type 2a)	calcium	[14]
Methylobacterium extorquens AM1	ethanol	META1_1139	ExaF (ADH type 2b)	lanthanide	[15]
Escherichia coli	glucose	P15877	m-GDH	calcium/ magnesium	[16]
Methylococcus capsulatus strain Bath	methanol	*7CE5	MxaF	calcium	[17]
Methylococcus capsulatus strain Bath	methanol	*4TQO	MxaF	calcium	[18]
Methylobacterium extorquens	methanol	*1W6S	MxaF	calcium	[19]
Methylophilus methylotrophus W3A1	methanol	*4AAH	MxaF	calcium	[20]
Hyphomicrobium denitrificans	methanol	*2D0V	MxaF	calcium	[21]
Methylophaga aminisulfidivorans MPT	methanol	*5XM3	MxaF	magnesium	[22]
Paracoccus denitrificans	methanol	*1LRW	MxaF	calcium	[23]
Methylobacterium extorquens	methanol	*1H4I	MxaF	calcium	[24]
Methylosinus trichosporium OB3b	methanol	ZP_06888489.1	MxaF	calcium	[25]
Pseudomonas putida KT2440	ethanol	*6ZCW	PedH (ADH type 2b)	praseodymium	[26]
Pseudogluconobacter saccharoketogenes	sorbose	*4CVB	Sorbose	calcium	[27]
Ketogulonicigenium vulgare	sorbose	*4MH1	Sorbose	calcium	[28]
Methylacidiphilum fumariolicum SolV	methanol	*6FKW	XoxF	europium	[29]
Methylacidimicrobium thermophilum AP8	methanol	*7O6Z	XoxF1	neodymium	[30]
Methylomirabilis oxyfera	methanol	DAMO_0134	XoxF1	lanthanum	[31]
Methylacidiphilum fumariolicum SolV	methanol	*4MAE	XoxF2	cerium	[32]
Methylotenera mobilis	methanol	Mmol_2048	XoxF4	lanthanum	[33]
Methylotenera mobilis	methanol	Mmol_1770	XoxF4	lanthanum	[33]
Methylomicrobium buryatense 5G	methanol	*6DAM	XoxF5	lanthanum	[34]
Methylobacterium extorquens	methanol	*6OC6	XoxF5	lanthanum	[35]
Bradyrhizobium sp. MAFF 211645	methanol	BAI77916.1	XoxF5	cerium	[36]
Bradyrhizobium diazoefficiens USDA 110	methanol	NP_772853.1	XoxF5	lanthanum	[37]
Methylobacterium radiotolerans JCM 2831	methanol	YP_001753202.1	XoxF5	lanthanum	[38]
Methylotenera mobilis	methanol	3409	XoxF5	lanthanum	[33]

Supplementary references

1. Keltjens JT, Pol A, Reimann J, Op den Camp HJM. PQQ-dependent methanol dehydrogenases: rare-earth elements make a difference. *Appl Microbiol Biotechnol* 2014; **98**: 6163–6183.
2. Matsutani M, Yakushi T. Pyrroloquinoline quinone-dependent dehydrogenases of acetic acid bacteria. *Appl Microbiol Biotechnol* 2018; **102**: 9531–9540.
3. Lavy A, McGrath DG, Matheus Carnevali PB, Wan J, Dong W, Tokunaga TK, et al. Microbial communities across a hillslope-riparian transect shaped by proximity to the stream, groundwater table, and weathered bedrock. *Ecol Evol* 2019; **9**: 6869–6900.
4. Diamond S, Andeer PF, Li Z, Crits-Christoph A, Burstein D, Anantharaman K, et al. Mediterranean grassland soil C-N compound turnover is dependent on rainfall and depth, and is mediated by genomically divergent microorganisms. *Nat Microbiol* 2019; **4**: 1356–1367.
5. Taubert M, Grob C, Howat AM, Burns OJ, Dixon JL, Chen Y, et al. XoxF encoding an alternative methanol dehydrogenase is widespread in coastal marine environments. *Environ Microbiol* 2015; **17**: 3937–3948.
6. Shimodaira H, Hasegawa M. Multiple comparisons of log-likelihoods with applications to phylogenetic inference. *Mol Biol Evol* 1999; **16**: 1114–1116.
7. Luo H. Evolutionary origin of a streamlined marine bacterioplankton lineage. *ISME J* 2015; **9**: 1423–1433.
8. Delmont TO, Pierella Karlusich JJ, Veseli I, Fuessel J, Eren AM, Foster RA, et al. Heterotrophic bacterial diazotrophs are more abundant than their cyanobacterial counterparts in metagenomes covering most of the sunlit ocean. *ISME J* 2022; **16**: 927–936.

9. Schön ME, Martijn J, Vosseberg J, Köstlbacher S, Ettema TJG. The evolutionary origin of host association in the Rickettsiales. *Nat Microbiol* 2022; **7**: 1189–1199.

10. Singleton CM, McCalley CK, Woodcroft BJ, Boyd JA, Evans PN, Hodgkins SB, et al. Methanotrophy across a natural permafrost thaw environment. *ISME J* 2018; **12**: 2544–2558.

11. Crystal Structure of Quinohemoprotein Alcohol Dehydrogenase from Comamonas testosteroni: STRUCTURAL BASIS FOR SUBSTRATE OXIDATION AND ELECTRON TRANSFER. *J Biol Chem* 2002; **277**: 3727–3732.

12. Chen Z-W, Matsushita K, Yamashita T, Fujii T-A, Toyama H, Adachi O, et al. Structure at 1.9 A resolution of a quinohemoprotein alcohol dehydrogenase from Pseudomonas putida HK5. *Structure* 2002; **10**: 837–849.

13. Toyama H, Chen Z-W, Fukumoto M, Adachi O, Matsushita K, Mathews FS. Molecular cloning and structural analysis of quinohemoprotein alcohol dehydrogenase ADH-IIG from Pseudomonas putida HK5. *J Mol Biol* 2005; **352**: 91–104.

14. Keitel T, Diehl A, Knaute T, Stezowski JJ, Höhne W, Görisch H. X-ray structure of the quinoprotein ethanol dehydrogenase from Pseudomonas aeruginosa: basis of substrate specificity. *J Mol Biol* 2000; **297**: 961–974.

15. Good NM, Vu HN, Suriano CJ, Subuyuj GA, Skovran E, Martinez-Gomez NC. Pyrroloquinoline Quinone Ethanol Dehydrogenase in Methylobacterium extorquens AM1 Extends Lanthanide-Dependent Metabolism to Multicarbon Substrates. *J Bacteriol* 2016; **198**: 3109–3118.

16. Cozier GE, Anthony C. Structure of the quinoprotein glucose dehydrogenase of Escherichia coli modelled on that of methanol dehydrogenase from Methylobacterium extorquens.

Biochem J 1995; **312 (Pt 3)**: 679–685.

17. Chan SI, Chuankhayan P, Reddy Nareddy PK, Tsai I-K, Tsai Y-F, Chen KH-C, et al. Mechanism of Pyrroloquinoline Quinone-Dependent Hydride Transfer Chemistry from Spectroscopic and High-Resolution X-ray Structural Studies of the Methanol Dehydrogenase from (Bath). *J Am Chem Soc* 2021; **143**: 3359–3372.
18. Culpepper MA, Rosenzweig AC. Structure and protein-protein interactions of methanol dehydrogenase from Methylococcus capsulatus (Bath). *Biochemistry* 2014; **53**: 6211–6219.
19. Williams PA, Coates L, Mohammed F, Gill R, Erskine PT, Coker A, et al. The atomic resolution structure of methanol dehydrogenase from Methylobacterium extorquens. *Acta Crystallogr D Biol Crystallogr* 2005; **61**: 75–79.
20. Xia Z, Dai W, Zhang Y, White SA, Boyd GD, Mathews FS. Determination of the gene sequence and the three-dimensional structure at 2.4 angstroms resolution of methanol dehydrogenase from Methylophilus W3A1. *J Mol Biol* 1996; **259**: 480–501.
21. Nojiri M, Hira D, Yamaguchi K, Okajima T, Tanizawa K, Suzuki S. Crystal structures of cytochrome c(L) and methanol dehydrogenase from Hyphomicrobium denitrificans: structural and mechanistic insights into interactions between the two proteins. *Biochemistry* 2006; **45**: 3481–3492.
22. Cao T-P, Choi JM, Kim SW, Lee SH. The crystal structure of methanol dehydrogenase, a quinoprotein from the marine methylotrophic bacterium Methylophaga aminisulfidivorans MP. *J Microbiol* 2018; **56**: 246–254.
23. Xia Z-X, Dai W-W, He Y-N, White SA, Mathews FS, Davidson VL. X-ray structure of methanol dehydrogenase from Paracoccus denitrificans and molecular modeling of its interactions with cytochrome c-551i. *J Biol Inorg Chem* 2003; **8**: 843–854.

24. Ghosh M, Anthony C, Harlos K, Goodwin MG, Blake C. The refined structure of the quinoprotein methanol dehydrogenase from Methylobacterium extorquens at 1.94 A. *Structure* 1995; **3**: 177–187.

25. Farhan Ul Haque M, Kalidass B, Bandow N, Turpin EA, DiSpirito AA, Semrau JD. Cerium regulates expression of alternative methanol dehydrogenases in Methylosinus trichosporium OB3b. *Appl Environ Microbiol* 2015; **81**: 7546–7552.

26. Wehrmann M, Elsayed EM, Köbbing S, Bendz L, Lepak A, Schwabe J, et al. Engineered PQQ-dependent alcohol dehydrogenase for the oxidation of 5-(hydroxymethyl)furoic acid. *ACS Catal* 2020; **10**: 7836–7842.

27. Rozeboom HJ, Yu S, Mikkelsen R, Nikolaev I, Mulder HJ, Dijkstra BW. Crystal structure of quinone-dependent alcohol dehydrogenase from Pseudogluconobacter saccharoketogenes. A versatile dehydrogenase oxidizing alcohols and carbohydrates. *Protein Sci* 2015; **24**: 2044–2054.

28. Han X, Xiong X, Jiang D, Chen S, Huang E, Zhang W, et al. Crystal structure of L-sorbose dehydrogenase, a pyrroloquinoline quinone-dependent enzyme with homodimeric assembly, from Ketogulonicigenium vulgare. *Biotechnol Lett* 2014; **36**: 1001–1008.

29. Jahn B, Pol A, Lumpe H, Barends TRM, Dietl A, Hogendoorn C, et al. Similar but Not the Same: First Kinetic and Structural Analyses of a Methanol Dehydrogenase Containing a Europium Ion in the Active Site. *Chembiochem* 2018; **19**: 1147–1153.

30. Schmitz RA, Picone N, Singer H, Dietl A, Seifert K-A, Pol A, et al. Neodymium as Metal Cofactor for Biological Methanol Oxidation: Structure and Kinetics of an XoxF1-Type Methanol Dehydrogenase. *MBio* 2021; **12**: e0170821.

31. Wu ML, Wessels JCT, Pol A, Op den Camp HJM, Jetten MSM, van Niftrik L. XoxF-type

methanol dehydrogenase from the anaerobic methanotroph 'Candidatus Methylomirabilis oxyfera'. *Appl Environ Microbiol* 2015; **81**: 1442–1451.

32. Pol A, Barends TRM, Dietl A, Khadem AF, Eygensteyn J, Jetten MSM, et al. Rare earth metals are essential for methanotrophic life in volcanic mudpots. *Environ Microbiol* 2014; **16**: 255–264.

33. Huang J, Yu Z, Chistoserdova L. Lanthanide-Dependent Methanol Dehydrogenases of XoxF4 and XoxF5 Clades Are Differentially Distributed Among Methylotrophic Bacteria and They Reveal Different Biochemical Properties. *Front Microbiol* 2018; **9**: 1366.

34. Deng YW, Ro SY, Rosenzweig AC. Structure and function of the lanthanide-dependent methanol dehydrogenase XoxF from the methanotroph Methylomicrobium buryatense 5GB1C. *J Biol Inorg Chem* 2018; **23**: 1037–1047.

35. Good NM, Fellner M, Demirer K, Hu J, Hausinger RP, Martinez-Gomez NC. Lanthanide-dependent alcohol dehydrogenases require an essential aspartate residue for metal coordination and enzymatic function. *J Biol Chem* 2020; **295**: 8272–8284.

36. Fitriyanto NA, Fushimi M, Matsunaga M, Pertiwiningrum A, Iwama T, Kawai K. Molecular structure and gene analysis of Ce3+ -induced methanol dehydrogenase of Bradyrhizobium sp. MAFF211645. *J Biosci Bioeng* 2011; **111**: 613–617.

37. Wang L, Suganuma S, Hibino A, Mitsui R, Tani A, Matsumoto T, et al. Lanthanide-dependent methanol dehydrogenase from the legume symbiotic nitrogen-fixing bacterium Bradyrhizobium diazoefficiens strain USDA110. *Enzyme Microb Technol* 2019; **130**: 109371.

38. Hibi Y, Asai K, Arafuka H, Hamajima M, Iwama T, Kawai K. Molecular structure of La3+-induced methanol dehydrogenase-like protein in Methylobacterium radiotolerans. *J*

Biosci Bioeng 2011; **111**: 547–549.
