# Supplementary figures and images for: Extensive and diverse lanthanide-dependent metabolism in the ocean
Source: ISME J. 2025 Apr 23;19(1):wraf057. doi: 10.1093/ismejo/wraf057 (PMC11996626; doi:10.1093/ismejo/wraf057)

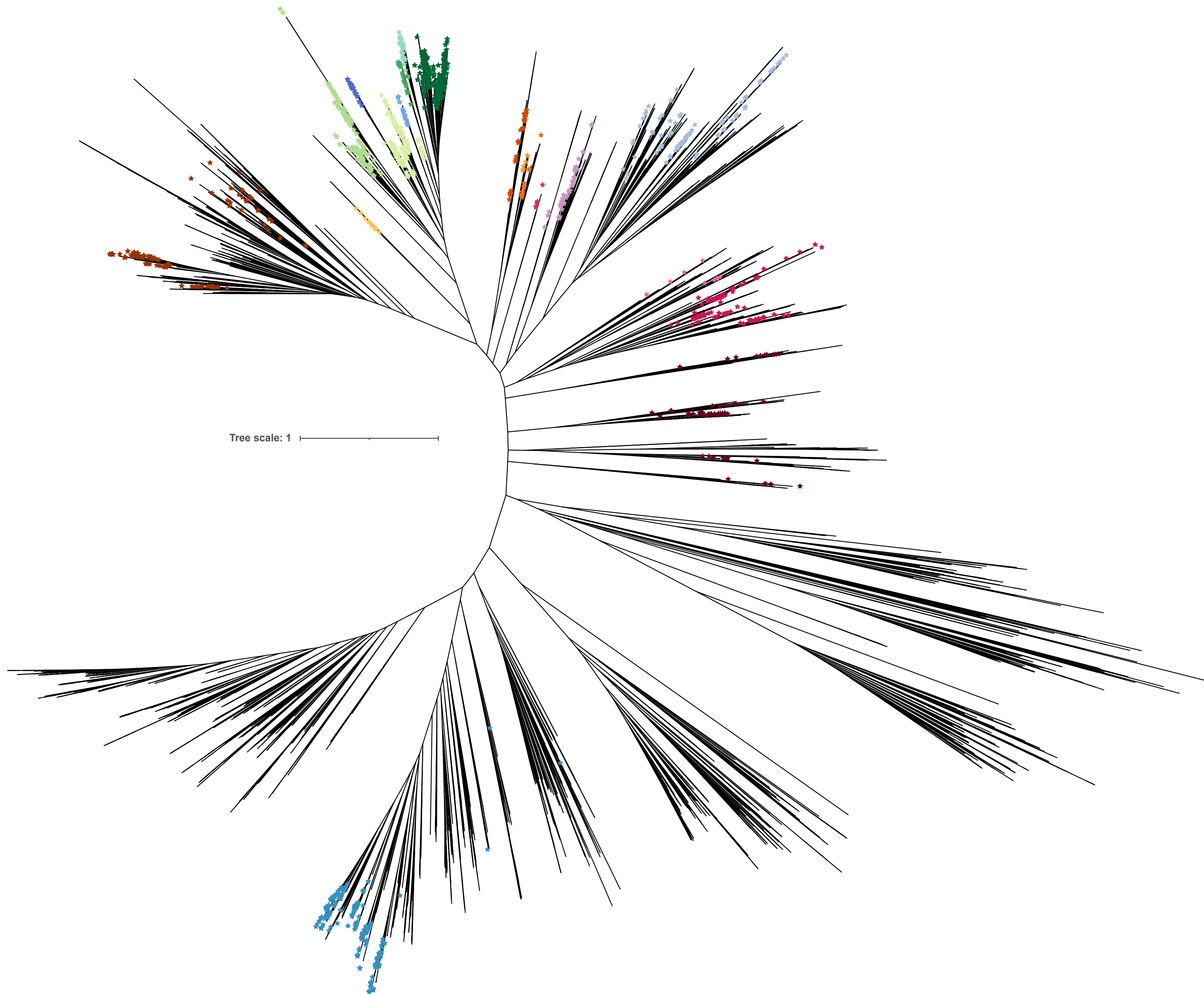

Tree scale: 1

Supplement: Fig_S2A_wraf057 [file fig_s2a_wraf057.pdf]

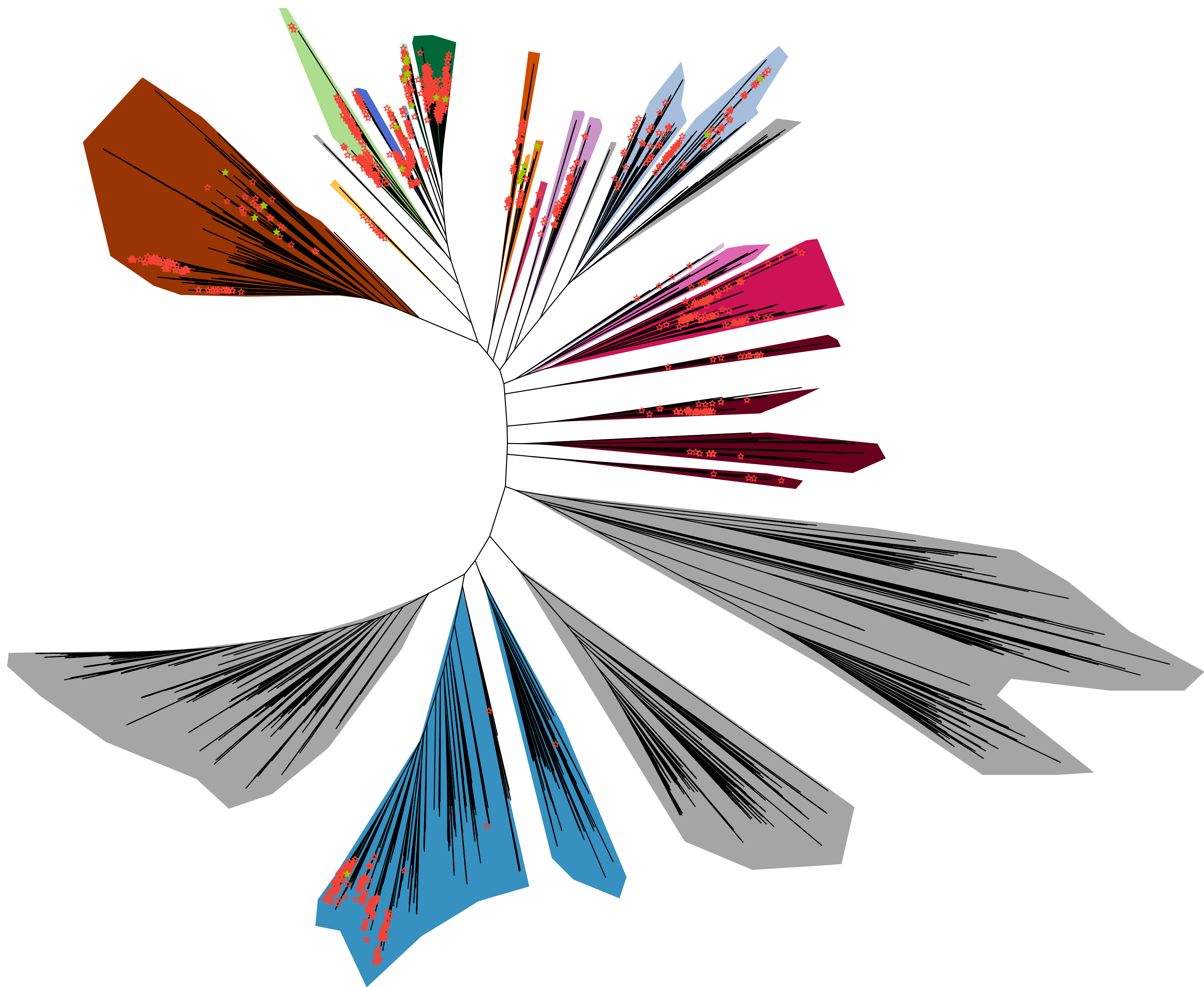

Supplement: Fig_S2B_wraf057 [file fig_s2b_wraf057.pdf]

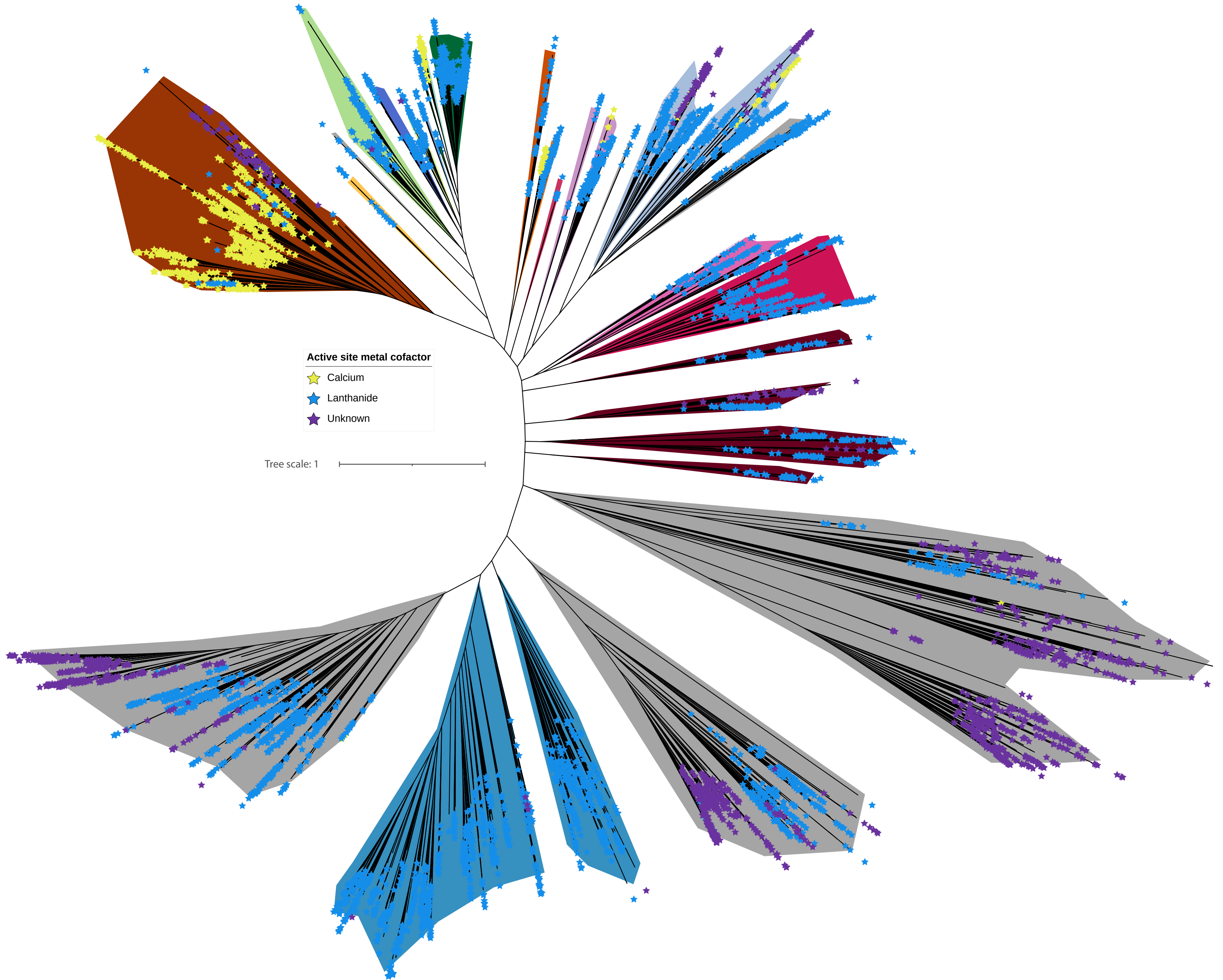

Supplement: Fig_S2C_wraf057 [file fig_s2c_wraf057.pdf]

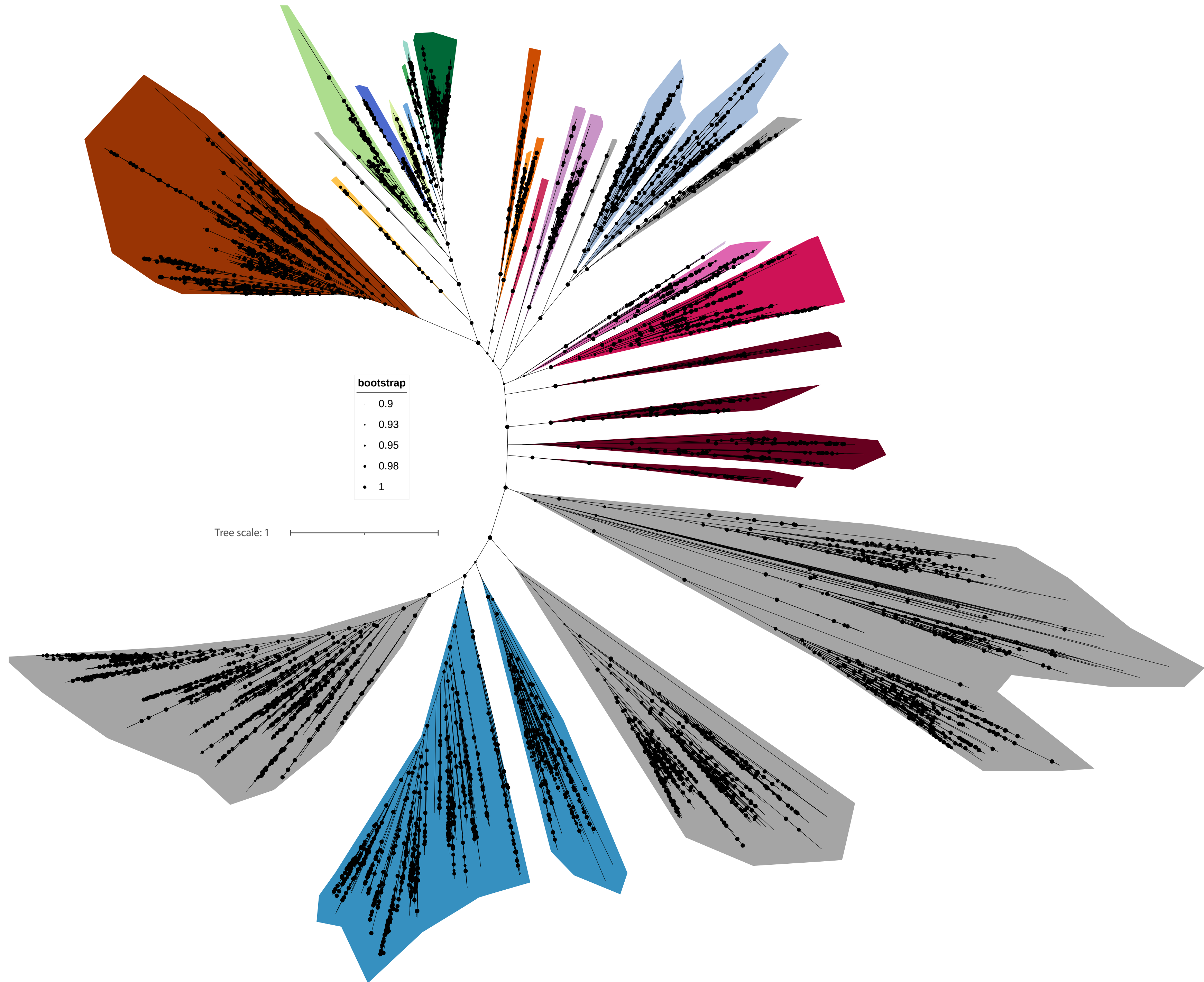

Supplement: Fig_S2D_wraf057 [file fig_s2d_wraf057.pdf]
